# Susceptibility-induced internal gradients reveal axon morphology and cause anisotropic effects in the diffusion-weighted MRI signal

**DOI:** 10.1038/s41598-024-79043-5

**Published:** 2024-11-28

**Authors:** S. Winther, H. Lundell, J. Rafael-Patiño, M. Andersson, J.-P. Thiran, T. B. Dyrby

**Affiliations:** 1 Department of Applied Mathematics and Computer Science, Technical University of Denmark, Kongens Lyngby, Denmark; 2 Danish Research Centre for Magnetic Resonance, Department of Radiology and Nuclear Medicine, Copenhagen University Hospital - Amager and Hvidovre, 2650 Copenhagen, Denmark; 3 Department of Health Technology, Technical University of Denmark, Kongens Lyngby, Denmark; 4 Signal Processing Laboratory (LTS5), École Polytechnique Fédérale de Lausanne (EPFL), Lausanne, Switzerland; 5 CIBM Center for Biomedical Imaging, Lausanne, Switzerland; 6 Radiology Department, Centre Hospitalier Universitaire Vaudois and University of Lausanne, Lausanne, Switzerland

**Keywords:** Biophysics, Brain, Computer science, Neuroscience

## Abstract

Diffusion-weighted MRI is our most promising method for estimating microscopic tissue morphology in vivo. The signal acquisition is based on scanner-generated *ex*ternal magnetic gradients. However, it will also be affected by susceptibility-induced *in*ternal magnetic gradients caused by interactions between the tissue and the static magnetic field of the scanner. With 3D in silico experiments, we show how internal gradients cause morphology-, compartment-, and orientation-dependence of spin-echo and pulsed-gradient spin-echo experiments in myelinated axons. These effects surpass those observed with previous 2D modelling corresponding to straight cylinders. For an ex vivo monkey brain, we observe the orientation-dependence generated only when including non-circular cross-sections in the in silico morphological configurations, and find orientation-dependent deviation of up to 17% for diffusion tensor metrics. Interestingly, we find that the orientation-dependence not only biases the signal across different brain regions, but also carries a sensitivity to the morphology of axonal cross-sections which is not attainable by the idealised theoretical diffusion-weighted MRI signal.

## Introduction

Diffusion-weighted MRI (dMRI) can reveal microscopic tissue morphology of the brain by measuring water diffusion properties within the tissue. The image contrast is induced by manipulating the phase coherence of nuclear spins with *ex*ternal magnetic gradients applied with the MR scanner. However, because different tissue components possess different magnetic susceptibilities, also *in*ternal magnetic gradients are induced in the tissue and will contribute to the image contrast. The internal gradients are morphology- and orientation-dependent, and correlate with the strength of the MR scanner’s $\textbf{B}_{\textbf{0}}$-field; thereby having a larger impact in the emerging ultra-high field scanners. The morphology-dependence gives rise to internal gradients across different scales; from the microscopic scale (e.g. between myelin and neighbouring tissue compartments) to the macroscopic scale (e.g. between different tissue regions). As diffusion lengths in a typical dMRI experiment is on the order of (10-100) $\mu \hbox {m}$, the internal gradients at the microscopic scale are particularly important. Meanwhile, gradients at the macroscopic scale will appear constant or linear within this diffusion range and are hence easily cancelled out. Some MRI sequences have been shown to successfully leverage functional and structural information contained by internal gradients to measure e.g. blood oxygenation^1^, tissue iron content^2^, myelination^3^, axon density^4^, axonal fiber directions^5–8^, and axonal orientation dispersion^9^.

dMRI sequences, on the contrary, are per design less sensitive to internal gradients, and effects of these are commonly disregarded. Early ex vivo studies found that orientation-dependent internal gradients have no measurable effect on the estimated diffusion anisotropy for optic garfish nerves and sciatic frog nerves at 2.35 T^10^, nor pig spinal cord at 4.7 T^11^. An in vivo study found no difference between ADC of human corpus callosum obtained from pulse-gradient spin-echo (PGSE) and the cross-term-reducing bipolar gradient pair (BGP) sequence at 1.5 T^12^. Recently, an in vivo study at 3 T^13^ found that fibre orientation w.r.t. $\textbf{B}_{\textbf{0}}$was responsible for up to 7% variance in the fractional anisotropy, when analysing white matter tracts across the whole brain and multiple subjects. Despite these findings, still no effect was found when analysing individual white matter fibres. Furthermore, in silico studies have investigated the effect of susceptibility on diffusion tensor (DT) metrics under variable diffusion times for 2D discs, randomly folded solid tubes, and random medium^14^and sample orientation for straight cylinders^15^. Nevertheless, given the dependence on morphology, orientation and field strength, further investigations for realistic axon morphologies and higher field strength hold the potential to provide novel insights into the underlying microstructure.

The most commonly applied sequence in dMRI is the pulsed-gradient spin-echo (PGSE)^16^. In the presence of internal gradients and diffusion beyond a lengthscale transcending the non-linearities of these gradients, the isolated spin-echo (SE) will be attenuated due to dephasing^17^. This is measured as a T2 effect. Moreover, because cross-terms arise between the external pulsed gradients and the internal gradients^16,18^, the PGSE signal becomes sensitive not only to diffusion in specific directions (as desired), but also to internal gradients in specific directions. These aspects may cause both the SE and PGSE signals from identical microstructure to deviate if oriented differently w.r.t. $\textbf{B}_{\textbf{0}}$.

Diffusion-weighting of the PGSE sequence can be signified by a *b*-vector. The desired b-vector $b_{des}$ is given from the user-specified scan sequence parameters. However, in the presence of internal gradients the effective b-vector $b_{eff}$ is a sum of contributions also from the internal gradients $b_{int}$, and from cross-terms between the internal and external gradients $b_{cross}$^16,18^. This means that $b_{eff}$will depend on the internal gradients of the given tissue, and thereby may reflect the morphology of the tissue. In addition, spatial variation in the internal gradient field would cause variance in the local dephasing and consequently contribute to non-mono-exponential attenuation of the signal. This would be reflected in higher-order diffusion terms^14^. Furthermore, because the impact of the internal gradients may be different across tissue compartments, the common normalization w.r.t. $S(b_{des}=0)$ may shift the weighting of different compartments.

Different strategies have been developed to correct for cross-terms in PGSE sequences. Cross-terms with internal gradients that appear linear over a characteristic diffusion length can be corrected by e.g. taking the geometric average of the signals from two b-vectors of opposite polarity^19,20^, using bipolar pulsed gradients on each side of the inversion pulse^21^, or numerical optimization scheme for general waveforms^18^. However, to cancel out cross-terms with internal gradients that appear nonlinear, more advanced sequences are required. A 13-interval STEAM sequence^22^ has proven capable - but is only practically applicable at long diffusion times. Effects of internal gradients in PGSE must therefore be addressed as part of the signal modelling. To do so, we need a better understanding of the mechanisms behind these internal gradients and their effects on dMRI signals.

In brain white matter, the main contribution to orientation-dependent internal gradients is believed to come from myelin due to its microscopic anisotropy^23,24^. Myelinated axons have commonly been approximated as infinitely long non-touching straight cylinders for microstructure modelling with dMRI^25^. For the special case of an infinitely long isolated straight coaxial cylinder, the susceptibility-induced gradients have been solved analytically; both when considering the susceptibility of myelin as isotropic^26^, and as anisotropic (i.e. modelling the polarity of the lipids within the myelin)^27,28^. In either case, gradients are induced only in the extra-axonal and myelin compartments, while the intra-axonal compartment is affected homogeneously; thereby leaving any intra-axonal SE signal unaffected. Furthermore, gradients are induced only when the orientation of the cylinder is non-parallel with the applied magnetic field.

From recent 3D synchrotron X-ray nano-holotomography (XNH) in monkey brain^29^, and 3D electron microscopy in rodent brains^30,31^, the morphology of myelinated axons has been found to demonstrate non-circular cross-sections, longitudinal diameter variations, and tortuous trajectories. Tortuous trajectories have previously been quantified by micro-dispersion^29^. These deviations from straight coaxial cylinders may induce internal gradients also in the intra-axonal compartment which have not been accounted for previously. More realistic axon morphology has been simulated in 2D based on ellipses and segmentations of electron microscopy images from mouse white matter^32^. These experiments do express internal gradients in the intra-axonal compartment. However, the 2D representation corresponds to infinitely long straight axons and thereby neglects all effects from the histology-documented longitudinal variations (diameter variation and tortuosity). Hence, any gradients that would be induced in the third dimension along the axons are not expressed.

In this work, we explore susceptibility-induced orientation-dependence of the SE and PGSE signals of myelinated axons at 7 T. We perform in silico experiments by integrating susceptibility-induced internal gradients in Monte Carlo diffusion simulations. With in silico experiments in individual axons, we study how different 3D morphological characteristics affect the orientation-dependence of the intra-axonal compartment. The characteristics have been quantified from XNH imaging of the corpus callosum (CC) of a vervet monkey brain^29^, and include non-circular cross-sections, longitudinal diameter variations, and trajectory variations. With in silico experiments in hexagonally packed helical axons, we study differences in orientation-dependence across the intra-axonal and extra-axonal compartments at different orientations. To compare our simulations with real MRI scans, we scan a cube of an ex vivo vervet monkey brain, and analyse the PGSE signals of the CC and the two cingula (CING-L and CING-R).

## Results

### Susceptibility-induced orientation-dependence of intra-axonal PGSE signal can distinguish morphological characteristics

**Fig. 1:**
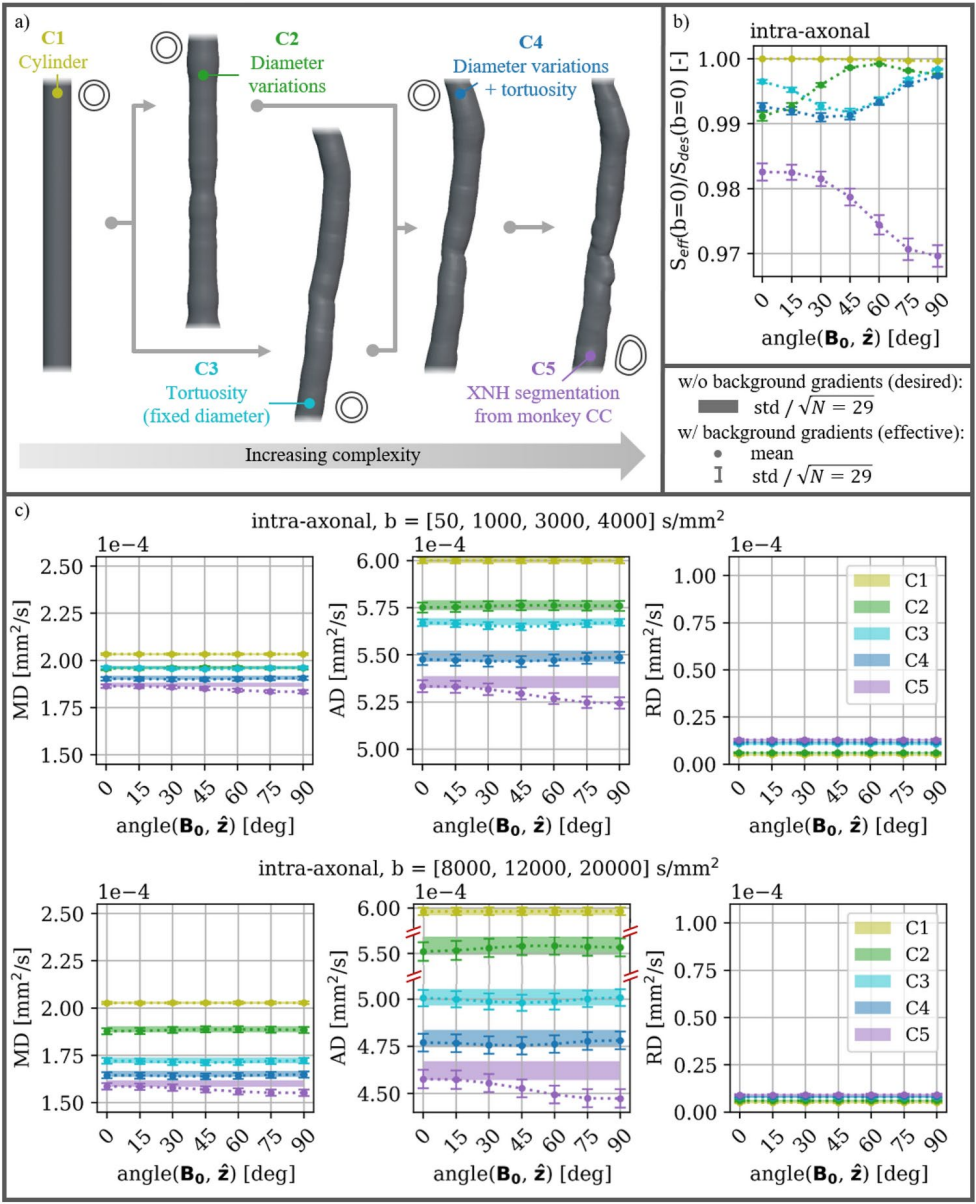
**a** Schematic overview of the XNH-informed axon configurations. Axon lengths are cropped to $40\,\mu \hbox {m}$ for visualization purposes. The morphological characteristics (mean diameter, longitudinal diameter variation, and trajectory) were quantified from 29 axons segmented from an XNH-volume, and expressed in the five configurations (C1-5). C5: segmentations of real axons, C1: straight with the mean diameter of the corresponding C5-axons, C2: straight with longitudinal diameter variations of the corresponding C5-axons, C3: mean diameter and tortuous trajectories of the corresponding C5-axons, and C4: longitudinal diameter variations and tortuous trajectories of the corresponding C5-axons. The overall orientation of each axon is aligned with $\hat{\textbf{z}}$. **b** The effect on the effective signal $S_{eff}$ measured relative to the desired signal $S_{des}$ at $b=0\,\hbox {s/mm}^2$ as a function of orientation w.r.t. $\textbf{B}_{\textbf{0}}$. This corresponds to an SE signal and thereby a T2 effect. **c** Mean diffusivity (MD), axial diffusivity (AD), and radial diffusivity (RD) obtained from DT fits to lower b-values (upper row) and higher b-values (lower row - note the discontinuous y-axis on the AD plot). Markers and error bars indicate mean and standard error of the metric for $S_{eff}$ over experiments in the 29 axons. Bands indicate the standard error of the metric centered around the mean for $S_{des}$. All y-axes have the same scale to emphasize the difference in the degree of orientation-dependence between the different metrics.

We integrated susceptibility-induced internal gradients into Monte Carlo diffusion simulations in the intra-axonal compartments of realistic axon morphologies. The morphologies were quantified from an XNH volume of the CC of a vervet monkey brain. Our analysis reveals that different morphological characteristics cause different trends of susceptibility-induced orientation-dependence (axon w.r.t. $\mathbf {B_{0}}$) for the PGSE signal. We analyze both $S(b_{des}=0)\,\hbox {s/mm}^2$ (corresponding to an SE signal and thereby a T2 effect), and diffusion tensor (DT) metrics fitted to b-values in different ranges. While the DT does not fully characterise the non-Gaussian diffusion in white matter, it does capture the principal anisotropy of the dMRI signal which is of our interest here; where the aim is to study relative changes in the anisotropic signal rather than exact values. The morphological characteristics (longitudinal diameter variation, longitudinal trajectory variation, and cross-sectional variation) are studied in five different configurations (C1-5) of increasing complexity; ranging from straight cylinders (Fig. 1a, C1), to real axons segmented from an XNH image (Fig. 1a, C5).

**Fig. 2:**
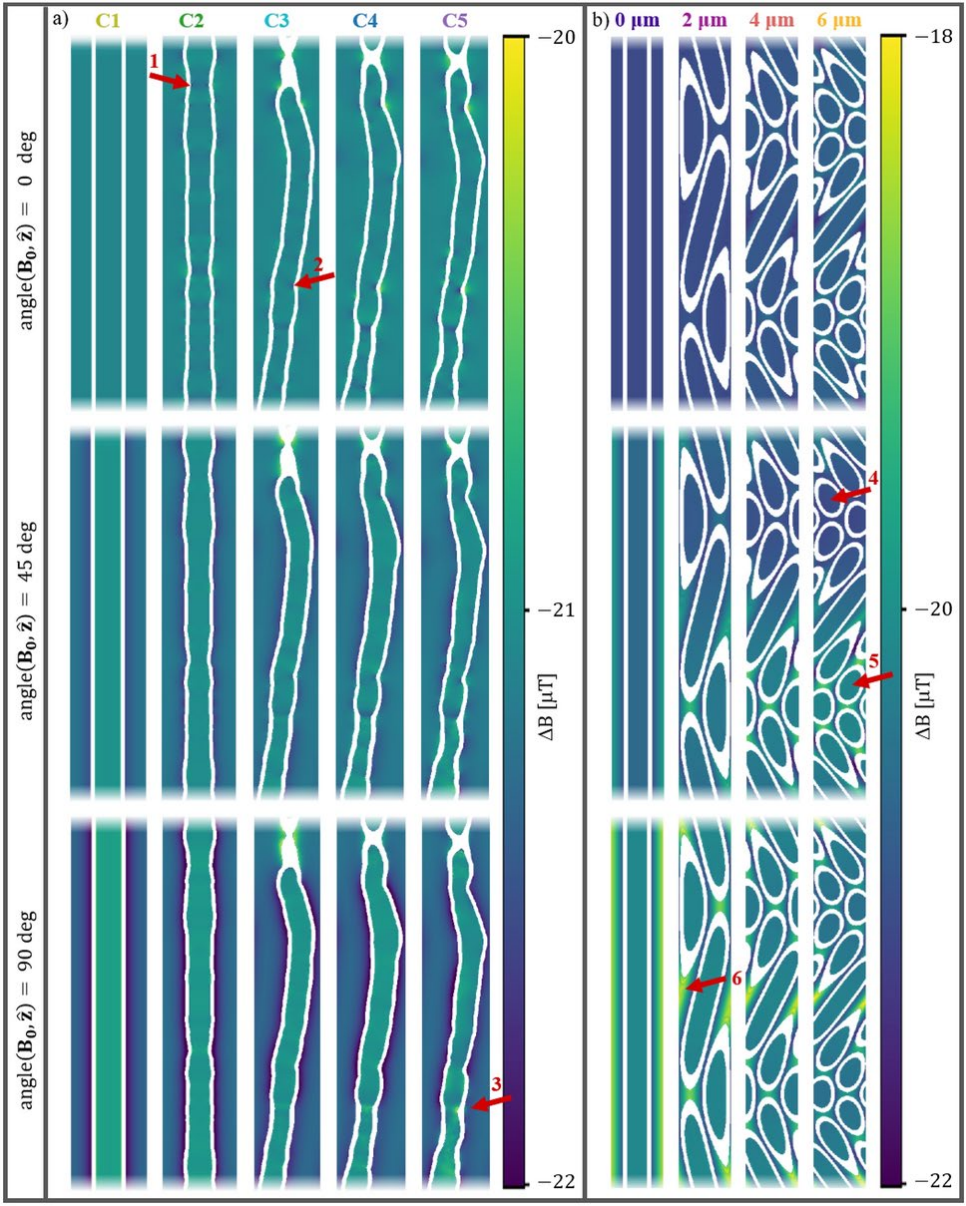
Cross-sections of the susceptibility-induced B-field changes at three different orientations between $\textbf{B}_{\textbf{0}}$ and the dominating orientation of the axons $\hat{\textbf{z}}$, i.e. $\text {angle}(\textbf{B}_{\textbf{0}},\hat{\textbf{z}})=[0, 45, 90]$ deg. To emphasize the field inhomogeneity in the compartments of interest (intra- and extra-axonal) the myelin compartment is hidden (shown in white), and the colour maps are cropped. **a** C1-5 axons (see Fig. 1.a). Cropped to $50\,\mu \hbox {m}$ for visualization purposes. **b** Helical axons (see Fig. 3a) yz-cross-sections showing an entire period $\lambda = 50\,\mu \hbox {m}$ of the periodic substrate. The arrows point to different examples of internal gradients. 1: caused only by diameter variation and most prominent at $\text {angle}(\textbf{B}_{\textbf{0}},\hat{\textbf{z}})=0$ deg. 2: caused only by tortuosity. 3: caused by the real axon morphology of the C5 and expressing a more complex pattern than what is observed at arrows 1 and 2. 4 and 5: attention to the difference in internal gradients induced along the helical axon for segments oriented differently w.r.t. $\textbf{B}_{\textbf{0}}$. 6: more prominent gradients in the extra-axonal compartment compared with the intra-axonal compartment. For a more detailed view of the B-field variations, please refer to the digital version of this article.

First, we explore how the internal gradients induced by the different morphological configurations (see Fig. 2) affect the signal at $b=0\,\hbox {s/mm}^2$. This is done by normalizing the effective signal $S_{eff}(b=0)$ (i.e. affected by internal gradients), w.r.t. the desired signal $S_{des}(b=0)$ (i.e. unaffected by internal gradients and corresponding to the number of particles). Figure 1b shows that each morphological configuration induces a unique trend of orientation-dependence. C1 expresses no orientation-dependence; in correspondence with the analytic model for straight coaxial cylinders^33^. C2 and C3 express the largest effect at $\text {angle}(\textbf{B}_{\textbf{0}}, \hat{\textbf{z}}) = 0$ deg and $\text {angle}(\mathbf {B_{0}}, \hat{\textbf{z}})=45$ deg respectively. C4 expresses a combination of the effects observed for C2 and C3; in correspondence with the characteristics of this configuration in fact being a combination of the two. C5 expresses the strongest effect at $\text {angle}(\textbf{B}_{\textbf{0}}, \hat{\textbf{z}})=90\,\hbox {deg}$, and is affected more than any other configurations at any orientation.

When fitting the DT to the PGSE signal, the same trends of orientation-dependence are observed for the mean diffusivity (MD) and the axial diffusivity (AD), while the radial diffusivity (RD) has no orientation-dependence or orientation-dependence much smaller than the variance of the signal (Fig. 1b vs. 1c). The orientation-dependences are strongest for AD, and weaker for MD which is a combination of AD and RD. The difference in the effect on AD and RD shows that the susceptibility-induced internal gradients are causing an anisotropic effect on the PGSE signal, and that this anisotropy is orientation-dependent. By comparing DT metrics fitted at lower b-values ($[50, 1000, 3000, 4000]\,\hbox {s/mm}^2$) with those fitted at higher b-values ($[8000, 12000, 20000]\,\hbox {s/mm}^2$), an increased orientation-dependence is seen at higher b-values (Fig. 1c).

The different axonal configurations have unique effects on the DT metrics fitted to $S_{des}(b)$ for both b-value regimes. AD decreases with increasing configuration number; in correspondence with increasing obstructions in the form of longitudinal morphological variation in the axial direction. RD increases with increasing configuration number; in correspondence with added axonal tortuosity in the radial direction. MD is dominated by AD due to the elongated axonal morphologies ($\lambda _1 \gg \lambda _2, \lambda _3$of the DT fits), and follows a similar decrease. Comparing DT metrics fitted at lower b-values with those fitted at higher b-values, shows a large decrease in the AD estimates for the morphological configurations expressing tortuosity. Meanwhile, C2 expresses only a small decrease, and C1 expresses no decrease. DT metrics for all morphological configurations show larger standard deviations at higher b-values. These results are in correspondence with previous work^29^.

### Susceptibility-induced orientation-dependence varies across tissue compartments and with the degree of axonal undulations

We now explore the effect of increasing the degree of undulation, and how the internal gradients can give rise to compartment-specific orientation-dependence, which in turn can cause orientation-dependent filtering of these tissue compartments. Undulation describes bending in a regular and repeating pattern, whereas tortuosity describes irregular bending. For this purpose, we construct numerical substrates of densely packed myelinated axons, where myelinated axons are constructed as coaxial helices on a hexagonal grid. The substrates resemble the C3 axons from the XNH-informed axon configurations (Fig. 1a), i.e. constant diameter and tortuous axon trajectories.

As the undulation amplitude increases, the direction of the first eigenvector of the DT fit ($\varvec{e}_1$) starts deviating from the axis of the helix ($\hat{\textbf{z}}$) (Fig. S1). Before including susceptibility-induced internal gradients in the signal, only the signal of the extra-axonal compartment has severe ($>5\,\hbox {deg}$) deviation between $\textbf{e}_{\textbf{1}}$ and $\hat{\textbf{z}}$. When internal gradients are integrated in the signal, we see orientation-dependent deviation between $\textbf{e}_{\textbf{1}}$ and $\hat{\textbf{z}}$ when $r_{helix} \ge 4 \mu \hbox {m}$. These deviations cause AD (the highest diffusivity of the DT) to not always be measured along the axial direction of these axons.

Without including internal gradients in the signal ($S_{des}$), the different degrees of undulation ($r_{helix}$) have different effects on the different DT metrics. AD decreases with $r_{helix}$, and RD increases with $r_{helix}$; in correspondence with increasing isotropy of the substrate. MD is dominated by AD when $\lambda _1>> \lambda _2, \lambda _3$, and follows a similar decrease with $r_{helix}$.

**Fig. 3:**
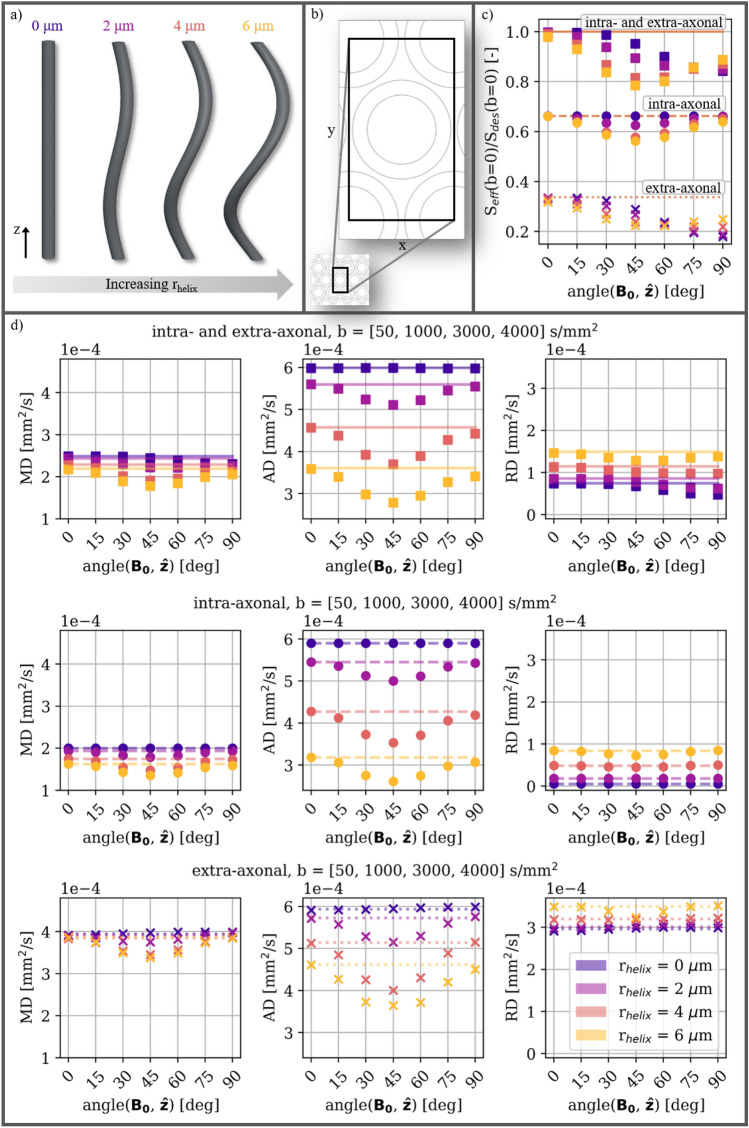
**a** Helical axons with radii $r_{axon}=1.90\,\mu \hbox {m}$, helical wavelength $\lambda =50\,\mu \hbox {m}$, and varying helical radii $r_{helix}$. **b** Cross-section of the unit voxel which is used to represent the hexagonally packed substrates. **c** The effect on the effective signal $S_{eff}$ measured relative to the desired signal $S_{des}$ at $b=0\,\hbox {s/mm}^2$ as a function of orientation w.r.t. $\textbf{B}_{\textbf{0}}$. This corresponds to an SE signal and thereby a T2 effect. Different markers indicate the DT metrics fitted to $S_{eff}(b)$ for the different compartments. Lines indicate the DT metrics fitted to $S_{des}(b)$. **d** Mean diffusivity (MD), axial diffusivity (AD), and radial diffusivity (RD) obtained from the DT model. Results for the combined intra- and extra-axonal compartments are shown in the upper row, for the intra-axonal compartment in the middle row, and for the extra-axonal compartment in the lower row. Markers indicate the DT metric fitted to $S_{eff}(b)$. Lines indicate the DT metric fitted to $S_{des}(b)$.

The T2 effect caused by the susceptibility-induced internal gradients is analyzed by normalizing the effective signal $S_{eff}(b=0)$ (i.e. affected by internal gradients), w.r.t. the desired signal $S_{des}(b=0)$ (i.e. unaffected by internal gradients). Figure 3c shows that the signals of the different tissue compartments have unique trends of dependence for both orientation and amplitude of the undulation. The substrate with $r_{helix} = 0.0 \mu \hbox {m}$ expresses no intra-axonal effect at any orientation, no extra-axonal effect at $\text {angle}(\textbf{B}_{\textbf{0}}, \hat{\textbf{z}}) = 0$ deg, and the largest extra-axonal effect at $\text {angle}(\textbf{B}_{\textbf{0}}, \hat{\textbf{z}}) = 90$ deg; in correspondence with our expectations for the analytic model for straight coaxial cylinders^33^. An intra-axonal effect arises with increasing $r_{helix}$. The extra-axonal compartment express minimal dependence on $r_{helix}$ at $\text {angle}(\textbf{B}_{\textbf{0}}, \hat{\textbf{z}}) = 0$ deg, increasing dependence at $\text {angle}(\textbf{B}_{\textbf{0}}, \hat{\textbf{z}}) = 45$ deg, and decreasing dependence at $\text {angle}(\textbf{B}_{\textbf{0}}, \hat{\textbf{z}}) = 90$ deg. The signal from the combined compartments shows a combination of the two individual compartments.

The DT metrics fitted to the individual compartments (Fig. 3d) have the strongest orientation-dependence for AD, while it is weaker for MD. RD demonstrates orientation-dependence only when $r_{helix} = 6.0\mu \hbox {m}$ (the largest $r_{helix}$ tested here). This is also the substrate with the largest deviation between $\textbf{e}_{\textbf{1}}$ and $\hat{\textbf{z}}$ (Fig. S1).

The DT metrics fitted to the combined signal of the intra- and extra-axonal compartments have noticeable orientation-dependent tissue-compartmental filtering due to the T2 effect at $b=0\,\hbox {s/mm}^2$. This is based on the signals from the individual compartments showing different degrees of signal loss in Fig. 3c. MD and RD have orientation-dependences that are different from those observed for the individual compartments (minimum value around 45 deg), while more similar to the T2 effects (described above).

### Susceptibility-induced orientation-dependence in ex vivo tissue

To test the orientation-dependence observed in silico, we performed ex vivo MRI scans of a tissue-block dissected from a perfusion-fixated vervet monkey brain, and scanned it at different orientations w.r.t. $\textbf{B}_{\textbf{0}}$. Two acquisitions were made; for b-values up to $4000\,\hbox {s/mm}^2$ in scan-series 1 (Fig. S2), and up to $20000\,\hbox {s/mm}^2$ in scan-series 2 (Fig. S3, S4, and 4). To assess the impact in different tissue compartments, we utilize that higher b-values attenuate the signal originating from faster diffusing nuclei and thereby the extra-axonal compartment. Thus, we fitted a DT model to different ranges of b-values. As validated in Fig. S5, the signal from the highest range ($b = [8000, 12000, 20000]\,\hbox {s/mm}^2$) originates from an isolated intra-axonal compartment. Meanwhile, the lower b-value ranges also reflect different degrees of the extra-axonal compartment. Orientation-dependence was quantified by ranking the best fitting trend (A-D) (see Sec. 4.4) based on the rescaled Akaike information criterion ($\Delta$AIC). Overall, we found that the degree of orientation-dependence (quantified in the fitting parameter $p_1$) and the magnitude (quantified in the fitting parameter $p_0$) of the DT metrics decrease with increasing range of b-values. Meanwhile, the degree of relative orientation-dependence ($p_1/p_0\cdot 100$) increases with the range of b-values (listed below). The degree of orientation-dependence in all ROIs is consistently largest for AD, and larger for CC than for CING. The difference in orientation-dependence of AD and RD indicates an anisotropic effect on the PGSE signal.

**Fig. 4:**
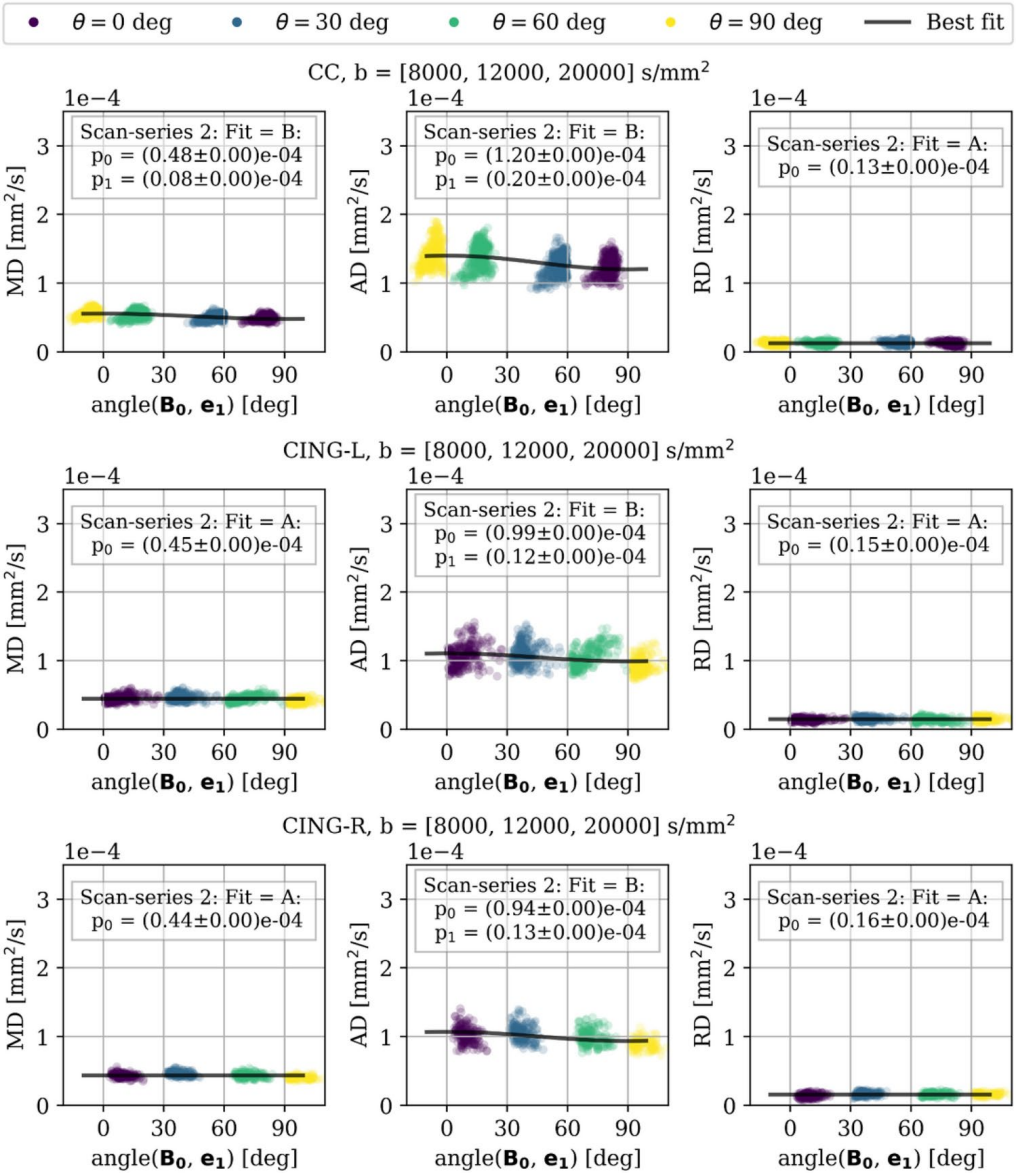
Scan-series 2. DT metrics fitted at high b-values ($[8000, 12000, 20000]\,\hbox {s/mm}^2$) for CC (upper row), CING-L (middle row), and CING-R (lower row) of an ex vivo monkey brain scanned at 7 T. Markers are coloured according to which orientation $\theta$ the scan was acquired at, and plotted along with the best fit based on lowest AIC-value. Orientation-dependence is observed for AD of all ROIs, and for MD of CC. The degree of orientation-dependence is stronger for CC than for CING-L and CING-R. The difference in effect on AD vs. RD shows that the PGSE signal is affected anisotropically. $\Delta$AIC and $\Delta$RMSE for all DT metrics and all fitted models are listed in Tab. S4.

At high b-values ($[8000, 12000, 20000]\,\hbox {s/mm}^2$, scan-series 2 in Fig. 4), where the signal is dominated by the intra-axonal compartment (validated in Fig. S5), CC demonstrates orientation-dependence of trend B for both MD and AD, and no orientation-dependence (trend A) for RD. This agrees with our in silico findings for the C5 axons which are also quantified from monkey CC. Meanwhile, CING-L and CING-R demonstrate orientation-dependence of trend B only for AD, and no orientation-dependence (trend A) for MD or RD. The estimated degrees of relative orientation-dependence are 16% for MD and 17% for AD in CC, and 12-14% for AD in CING.

At intermediate b-values ($[4000, 8000, 12000]\,\hbox {s/mm}^2$, scan-series 2 in Fig. S4), CC, CING-L, and CING-R demonstrates orientation-dependence of trend B for both MD and AD, and no orientation-dependence (trend A) for RD. However, both trend A and B show substantial support for MD in CING-R with $\Delta \hbox {AIC}<2$, and also trend B show some support for MD in CING-L with $4 \le \Delta AIC \le 7$. The estimated degrees of relative orientation-dependence are 15% for MD and 13% for AD in CC, and 12-12% for MD and 9-10% for AD in CING.

At low b-values ($[50, 1000, 3000, 4000]\,\hbox {s/mm}^2$, scan-series 2 in Fig. S3 and scan-series 1 in Fig. S2), all ROIs demonstrate orientation-dependence of trend B for both MD and AD, and no orientation-dependence (trend A) for RD. In scan-series 2, the estimated degrees of relative orientation-dependence are 7% for MD and 8% for AD in CC, and 5-5% for MD and 5-6% for AD in CING.

## Discussion

With in silico experiments, we show that the susceptibility-induced orientation-dependence trends for the intra-axonal SE and PGSE signals are specific to the morphological characteristics expressed in an axon population. Both the cross-sectional and longitudinal morphological variations prove to impact the orientation-dependence. Compared to previous 2D modelling^32^, our 3D modelling thereby provides additional insight into susceptibility-induced orientation-dependence of myelinated axons. Interestingly, our findings show potential for a sensitivity to cross-sectional morphology of myelinated axons. This sensitivity is not attainable with the theoretical dMRI signal ($S_{des}$), due to the limited spatial resolution averaging over an ensemble of axons, and thereby smearing out the signal fromthe individual axons. However, we demonstrated that the sensitivity to cross-sectional morphology can be obtained by leveraging the susceptibility-induced orientation-dependence. Furthermore, we show that the intra- and extra-axonal compartments can be affected differently by these internal gradients, and thereby cause orientation-dependent filtering of the signals from those compartments.

With ex vivo experiments in monkey (CC, CING-L, and CING-R), we reproduced the orientation-dependence trend observed only for the in silico axon population expressing the full range of morphological characteristics (longitudinal diameter variations, tortuosity, and cross-sectional variation - i.e. real axons segmented from XNH volumes). Hence, our results contradict the more simple representations of myelinated axons without longitudinal morphological variation.

We show that AD demonstrates orientation-dependence while RD remains stable. We attribute this anisotropic effect of the PGSE signal to arise from the cross-terms between the external and internal gradients. Previously, orientation-dependence of dMRI has only been documented across different white matter regions where differences in microstructure across regions cannot be ruled out as a contributing factor^8,15,34^.

The orientation-dependence observed in this study is further reason to take caution when comparing signals from different regions of myelinated axons. This is relevant both for axon populations oriented differently w.r.t. $\textbf{B}_{\textbf{0}}$, and for axon populations expressing different morphological characteristics. If effects of internal gradients are not incorporated in the applied modelling, the certainty of the model estimates may be limited due to the orientation-dependence. This becomes only more relevant with higher $\textbf{B}_{\textbf{0}}$ strengths.

### Linking axon morphology characteristics to orientation-dependence

With in silico experiments, we show that different morphological characteristics of myelinated axons (longitudinal diameter variations, tortuous trajectories, and cross-sectional eccentricity) introduce different susceptibility-induced orientation-dependence trends of the intra-axonal SE and PGSE signals. The different morphological configurations (C1-5) of increasing complexity are seen in Fig. 1a. In this section, we focus on the results for SE experiments shown in Fig. 1b where the trends are observed most clearly.

C1 not expressing any orientation-dependence of intra-axonal signals is explained in the analytic solution for the coaxial cylinder^26–28^, where no gradients are induced in the intra-axonal compartment.

C2 expressing the largest effect at $\text {angle}(\textbf{B}_{\textbf{0}}, \hat{\textbf{z}}) = 0$ deg may be explained by Eq. 4, where the morphological variations in the direction of $\textbf{B}_{\textbf{0}}$ have the greatest influence on the resulting B-field. Because C2 has circular cross-sections, the morphology with the greatest influence when approaching $\text {angle}(\textbf{B}_{\textbf{0}}, \hat{\textbf{z}}) = 90$ deg will resemble the coaxial cylinder (i.e. C1). Meanwhile, the morphology with the greatest influence when approaching $\text {angle}(\textbf{B}_{\textbf{0}}, \hat{\textbf{z}}) = 0$ deg is the longitudinal diameter variations.

C3 and the helixes having the largest effect at $\text {angle}(\textbf{B}_{\textbf{0}}, \hat{\textbf{z}}) = 45$ deg may be explained by the distribution of angles between $\textbf{B}_{\textbf{0}}$ and discretised longitudinal axon segments ($\text {angle}(\textbf{B}_{\textbf{0}}, \text {axon segments})$) (see Fig. S6). When $\text {angle}(\textbf{B}_{\textbf{0}}, \hat{\textbf{z}}) = 0$ deg, the distribution of $\text {angle}(\textbf{B}_{\textbf{0}}, \text {axon segments})$ is relatively narrow due to the axons’ overall alignment with $\hat{\textbf{z}}$. When $\text {angle}(\textbf{B}_{\textbf{0}}, \hat{\textbf{z}}) = 90$ deg, the distribution of $\text {angle}(\textbf{B}_{\textbf{0}}, \text {axon segments})$ is again relatively narrow due to the axial symmetry around 90 deg. When $\text {angle}(\textbf{B}_{\textbf{0}}, \hat{\textbf{z}}) = 45$ deg, $\text {angle}(\textbf{B}_{\textbf{0}}, \text {axon segments})$ has the widest distribution extending all way from $\text {angle}(\textbf{B}_{\textbf{0}}, \hat{\textbf{z}}) = 0$ deg to 90 deg. This orientation thereby captures the largest variety of induced field inhomogeneity.

C4 demonstrating a combination of the effects of C2 and C3, may be explained by the morphological characteristics expressed in C4 being the combination of those expressed in C2 and C3.

Interestingly, the trend of C5 stands out significantly from that of C4. The axons of C5 are the actual segmentations of real axons, and the only difference from C4 is non-circular cross-sections. Recall that axons of the configurations C1-4 all have synthesised circular cross-sections. C5 stands out by demonstrating the largest effect at $\text {angle}(\textbf{B}_{\textbf{0}}, \hat{\textbf{z}}) = 90$ deg, a larger degree of orientation-dependence overall, and a significant negative offset of the signal compared with C1-4. These effects may be explained by the non-circularity which due to the associated irregularities induces increased field inhomogeneity at all orientations, and especially at $\text {angle}(\textbf{B}_{\textbf{0}}, \text {axon segments}) = 90$ deg. For C1-4 the morphology with the greatest influence when approaching $\text {angle}(\textbf{B}_{\textbf{0}}, \hat{\textbf{z}}) = 90$ deg will resemble the coaxial cylinder due to their circular cross-sections.

Furthermore, the spatial variations in susceptibility associated with the non-circular cross-sections occur on a short length scale compared to the variations due to diameter variations and tortuosity, and the associated phase accumulations are therefore less likely to be cancelled out. From analytic modelling^33^ and the C1 axons, we know that the morphology of a coaxial cylinder results in no intra-axonal orientation-dependence of the signal. When acquired at a single orientation w.r.t. $\textbf{B}_{\textbf{0}}$, the dMRI signal is not sensitive to non-circular cross-sections due to the limited spatial resolution averaging over an ensemble of axons. However, by acquiring scans at multiple orientations, the orientation-dependence trend appears to be sensitive to non-circular cross-sections. Future work will focus on delineating how the cross-sectional morphology affects the susceptibility-induced orientation-dependence.

### Ex vivo orientation-dependence trends deviate from in vivo observations

We show that the orientation-dependence trend of MD and AD observed in the corpus callosum and cingula of an ex vivo monkey brain corresponds to that observed for the numerical C5 axons (XNH-segmentation from an ex vivo monkey corpus callosum expressing longitudinal diameter variation, tortuosity, and non-circular cross-sections).

A recent study by Kleban et al^13^. examined the impact of head orientation w.r.t. $\textbf{B}_{\textbf{0}}$ on DT metrics pooled from white matter fibres across five in vivo human brains at 3 T. A tiltable head coil (originally designed to increase patient comfort in the scanner) was re-purposed for acquiring scans at different orientations w.r.t. $\textbf{B}_{\textbf{0}}$^35^. While correlations were found between DT metrics and the orientation for specific white matter regions (Fig. 4 in^13^), it was not possible to interpret any orientation-dependence trends - likely due to the limited 18 deg difference in orientations w.r.t. $\textbf{B}_{\textbf{0}}$. Instead, trends were observed for voxels pooled from across different white matter regions (Fig. 3 in^13^). Although these are not specific to the morphology and compartmental volume fraction of specific white matter regions, a comparison to our results is relevant. Interestingly, these trends deviate from the trends observed in our study. Instead of resembling the numerical C5 axons, the trends observed by Kleban et al. resemble that of the numerical C4 axons (i.e. axons expressing longitudinal diameter variation and tortuosity but circular cross-sections rather than non-circular cross-sections).

The studies have several differences. Most prominent is that one study was performed in vivo and the other ex vivo. Tissue fixation is necessary in order to conserve the tissue ex vivo. Although this process intends to minimise changes in microstructural properties, some change is inevitable (see Sect. 3.7.3). Hence, the deviation between ex vivo and in vivo orientation-dependence trends could indicate a change in the morphological features of myelinated axons caused by ex vivo tissue processing.

Undoubtedly, this motivates further research due to the potential impact on the transfer of morphological observations between ex vivo and in vivo studies. Not only for MRI but also for other ex vivo imaging modalities.

### Orientation-dependent filtering of tissue compartments

We show that susceptibility-induced internal gradients can give rise to compartment-specific effects, and thereby orientation-dependent filtering (or weighting) of the tissue compartments. The simplest example of this is seen for the substrate with $r_{helix}=0\,\mu \hbox {m}$ (i.e. straight coaxial cylinders) in Fig. 3. Although neither the intra-axonal nor the extra-axonal compartments demonstrate orientation-dependence of the RD, the combined signal does (Fig. 3d, rightmost column). This can be explained by orientation-dependent filtering of the signals at $b=0\,\, \hbox {s/mm}^2$ (Fig. 3c). While the signal of the intra-axonal compartment does not appear affected by internal gradients at $\text {angle}(\textbf{B}_{\textbf{0}}, \hat{\textbf{z}}) = 90$ deg, the extra-axonal compartment does. Hence, at this orientation, the combined signal will have a smaller contribution from the extra-axonal compartment compared with the orientation $\text {angle}(\textbf{B}_{\textbf{0}}, \hat{\textbf{z}}) = 0$ deg. Since the extra-axonal RD is higher than the intra-axonal RD, this results in a lower combined RD. This generalizes to the substrates with $r_{helix}>0\,\mu \hbox {m}$. We expect this concept to generalise to intra- and extra-axonal compartments with other morphological configurations.

### With great b-value comes great filtering

We show that AD of the intra-axonal signal decreases through the morphological configurations C1-5; in correspondence with increasing obstructions in the axial direction caused by the morphological characteristics (Fig. 1c). The dependence on obstructions in the axial direction increases significantly at higher b-values. Especially the configurations expressing tortuosity (i.e. C3-5) are affected. For $S_{des}(b)$ this is solely due to the higher b-values filtering out the faster diffusing components (i.e. sections of the axons parallel with the external gradient). In continuation, this leads to filtering of which morphological aspects are represented in the signals. Morphological aspects leading to lower diffusivity will thereby dominate the signal. Because such aspects are likely to be correlated with characteristics of internal gradients, we expect $S_{eff}(b)$ to likewise reflect a subset of internal gradients and thereby influence the filtering. Furthermore, the stronger external gradients at the higher b-values will lead to a stronger influence by the cross-terms between the internal and external gradients. Since the internal gradients are anisotropic, so are the cross-terms. This may lead to additional directional filtering of the signal (discussed further in Sec. 3.5).

Our ex vivo experiments reproduced the orientation-dependence trend observed only for the in silico axon population expressing the full range of morphological characteristics (longitudinal diameter variations, tortuosity, and non-circular cross-sections). We observe an increase in orientation-dependence at higher b-values where the signal is dominated by contributions of lower diffusivity, and therefore assumed to be dominated by the intra-axonal compartment. This may indicate that the subset of internal gradients associated with the contributions of lower diffusivity causes more pronounced susceptibility-induced effects. This is likely a consequence of a higher degree of non-linearity of these internal gradients. However, disentangling the combined effects of internal and external gradients is a complex matter.

### Anisotropy of cross-term contributions

With both in silico and ex vivo experiments, we show that the PGSE signal in white matter is affected anisotropically by susceptibility-induced internal gradients. How the internal gradients affect the PGSE signals depends on the anisotropic cross-terms between internal and external gradients, and the anisotropic apparent diffusivity^16,18^. The DT assumes the signal to follow $\exp (-b_{des} D)$^36,37^, when in fact the signal follows $\exp (-b_{eff} D)$. This means that any diffusion weighting deviating from $b_{des}$ will be reflected in the fitted variable *D*. Hence, any additional diffusion weighting from internal contributions will lead to an underestimation of *D*in the given direction - and vice versa. A spatially variant internal gradient field may provide a spatially dependent augmented dephasing of spins. Variance in local dephasing effects may further provide a non-mono-exponential attenuation^14^. If we consider the hexagonally packed axons with $r_{helix} = 0\,\mu \hbox {m}$ (Fig. 3), we see that whenever the axons are not parallel with the applied field, internal gradients will be induced in the extra-axonal space. This is in correspondence with analytic solutions for the straight coaxial cylinder^26–28^. The gradients will be induced perpendicular to the axis of the axons, meaning that any line parallel with the axon will be an isoline. As expected, the AD (in this case modelling diffusion along the isolines of the internal gradients) shows no impact from internal gradients. When $r_{helix}> 0\,\mu \hbox {m}$ the isolines of the internal gradients are no longer straight and parallel with the AD. In this case, the degree of orientation-dependence increases with $r_{helix}$. The RD, however, is modelling diffusion perpendicular to the isolines of the internal gradients for $r_{helix} = 0\,\mu \hbox {m}$, but still shows no impact by internal gradients. We believe this is due to the much lower diffusivity in the plane perpendicular to the axons, as this allows much less probing of the internal gradients.

### Expected impact of susceptibility-induced biases in dMRI modelling

We studied the orientation-dependence of signals from myelinated axons. Naturally, demyelination^38–40^would affect the results. While demyelination is best detected with other MRI modalities such as T2 and FLAIR^41,42^, and the primary effect is assumed to be on the extra-axonal diffusion processes and increased exchange across compartments, our findings suggest that also intra-axonal DT metrics can be affected directly through the change in internal gradients.

We show that axon diameter variations can cause significant intra-axonal signal loss when the axons are oriented parallel with $\textbf{B}_{\textbf{0}}$ (C2 in Fig. 1). Axonal beading (and thereby amplified diameter variations) has been observed in various diseases such as multiple sclerosis^38^, ischemic stroke^43^, and traumatic brain injury^44^. We expect such pathomorphology to influence the orientation-dependence trends of $S_{eff}(b=0)$ and the AD.

We document morphology- and orientation-dependence of AD. Several models for PGSE signals are based on a fixed value for the axial diffusivity. This may lead to a bias across different brain regions because the different regions express different axon morphologies and orientations. By assuming a fixed value for the axial diffusivity, the susceptibility-induced variation of axial diffusivity may therefore be reflected in the additional model parameters instead. Examples are axon diameter estimations with ActiveAx^45^and with SMT^46^, and orientation dispersion and density estimates with NODDI^47^. However, some tolerance to imprecise fixation of axial diffusivity has been documented for the SMT model^48^.

We study predominantly parallel axons. For brain regions with a high degree of global dispersion, we expect the orientation-dependence of the SE and PGSE signals to cancel out. This is in agreement with findings for the gradient-echo signal^9^.

### Limitations

#### Field strength and sequence-parameters

The degree of susceptibility-induced internal gradients increases with increasing field strength. Here, we studied the effects at 7 T. We expect more severe orientation-dependence at higher field strengths. With a tendency of moving towards higher field strength due to the increased SNR, understanding the effects of internal gradients become only more relevant.

We show the results for one set of PGSE sequence-parameters. However, the results for both $S_{des}(b)$^29,49,50^ and $S_{eff}(b)$^14^ will depend on the choice of sequence-parameters.

#### In silico experiments

Due to the high memory usage when computing the susceptibility-induced internal gradients at adequate spatial resolution, our in silico experiments were limited to only very small unit voxels. This did not enable representation of a realistic packing of the axons. Chen et al^51^. showed that internal gradients from hexagonal packing cause faster signal loss for the gradient-echo compared to random packing in 2D simulations. We expect a similar effect for the hexagonal packing investigated here in 3D. A more realistic packing could be obtained by assuming the interaction between the fields of individual axons to be negligible compared to the interaction between the individual axon and $\textbf{B}_{\textbf{0}}$. This could be implemented by computing the internal gradients of individual axons, and then adding these contributions to an arbitrary grid while allowing super-position only in the extra-axonal compartment.

The XNH-informed axon configurations were based on 29 axons from the same tissue sample. Due to the low signal-to-noise ratio of the XNH volumes, a robust manual segmentation proved challenging for axons with mean diameters smaller than $2 \mu$. Hence, the segmented axons are not representative of the underlying distribution w.r.t. diameter^29^, and the diameter variation between the axons considered here, may therefore be more limited than that of the true underlying distribution. Higher morphological variation among the axons may lead to higher variation in the signals shown in Fig. 1.

Myelin is believed to be the main contributor to internal gradients in white matter^23^. We studied the effect of different axon and myelin morphology but did not consider effects from nodes of Ranvier. Such variations in myelin are expected to induce further inhomogeneity. We neglected the anisotropic organisation of the myelin lipids. The anisotropic organisation can potentially give rise to additional dependence on the orientation between axons and $\textbf{B}_{\textbf{0}}$^32^, along with dipolar effects^28^, which could contribute to a magic angle effect^35^. We approximated the susceptibility values for myelin, intra-axonal and extra-axonal compartments based on those of biological fat ($-7.79\cdot 10^-{-6}$) and water ($-9.04\cdot 10^-{-6}$)^52,53^. Deviation in the differences of susceptibility across compartments would influence the results. Larger differences would cause stronger field inhomogeneity and vice versa^54^. measured the susceptibility of pure phospholipids DPPC to $-9.68\cdot 10^-{-6}$. Using this approximation would result in weaker field inhomogeneity. A change from positive to negative difference is expected to inverse the field. However, the resulting effect on the phase accumulation would be the same.

Due to the differences in the internal gradients induced in the individual tissue compartments, we expect that exchange across the myelin has an impact on the effective signal. We did not model such effects here.

#### Ex vivo experiments and relation to in vivo systems

Several aspects make it infeasible to directly transfer our ex vivo findings to in vivo predictions. The complete impact of tissue fixation is unknown, but both microstructural and diffusion properties undergo changes compared to when the tissue is in its in vivo state. The tissue scanned here, was perfusion fixated with formaldehyde which is the most well-established chemical fixation agent for MRI^55^. However, chemical fixation with aldehydes may in some cases compromise the myelin structure by loosening and splitting of adjacent membrane layers^56^. This would change the bulk susceptibility properties of the myelin, and hence, the susceptibility-induced internal gradients. Chan et al^57^. found contributions of MR phase contrast to differ substantially between in vivo and ex vivo scans. Although fractional anisotropy is reported as stable under these conditions, D’Arceuil et al^58^. report up to 80% decrease in the apparent diffusion coefficient in white matter due to formalin fixation. Birkl et al^59^. report a 4% decrease in water content, and a >25% decrease in T2 values in white matter due to formalin fixation. By rinsing the tissue with saline prior to scanning (as done here), T2 values can be partly restored^60^. Based on these deviations between in vivo and ex vivo tissue, we expect stronger susceptibility effects in vivo due to 1) higher diffusivity, 2) longer TEs, and 3) more structured myelin.

## Methods

### Monkey brain tissue

One brain was prepared as part of the synchrotron experiment described in Andersson et al^29^. to image axons in nanometer image resolution. The second brain was used for the dMRI experiments. The tissue comes from two perfusion fixed female and age matched (32 month) vervet (Chlorocebus aethiops) monkey brains, obtained from the Montreal Monkey Brain Bank. The monkeys, cared for on the island of St. Kitts, had been treated in line with a protocol approved by The Caribbean Primate Center of St. Kitts. The brains had previously been stored and prepared according to Dyrby et al^61^.

### In silico experiments

In silico DWI experiments were carried out for two branches of axon-mimicking numerical substrates: 1) isolated axons expressing different combinations of morphological characteristics quantified from axons previously segmented from synchrotron X-ray nano-holotomography (XNH) volumes^29^, and 2) hexagonally packed helical axons (coaxial cylinders). Monte Carlo diffusion simulations were carried out with surface mesh representations of the numerical substrates. For each mesh representation we generated a three-compartment volume segmentation (intra-axonal, extra-axonal and myelin) as a voxelized representation from where we computed the susceptibility-induced internal gradients. The internal gradients were integrated into the Monte Carlo simulations by including the phase accumulation caused by the internal gradients when computing the DWI signal.

#### XNH-informed axon configurations (intra-axonal compartment)

To study how different morphological characteristics of axons affect the susceptibility-induced internal gradients and the related SE and PGSE signals, we conducted simulations for 29 segmentations of real axons, and four different configurations of morphological characteristics quantified from each of these (Fig. 1a). The quantified characteristics are mean axon diameter, longitudinal diameter variation, and axonal trajectory. Expressed in the five configurations (C1-5) we have C5: segmentations of real axons, C1: mean diameter with straight axon trajectories (cylinders), C2: longitudinal diameter variations with straight axon trajectories, C3: mean diameter and tortuous axon trajectories, and C4: longitudinal diameter variations and tortuous axon trajectories. All axons are aligned with the z-axis.

The axons were segmented from a 3D XNH volume of the splenium of a 32-month old vervet monkey brain. The volume was acquired with 75 nm isotropic voxel size and $153.6\,\upmu \text {m}$ cylindrical field-of-view at beamline ID16A of the European Synchrotron. Fifty-four axons with a minimum length of $120\,\upmu \text {m}$ were semi-manually segmented in the native image resolution, after which their trajectories were determined, allowing for a quantification of longitudinal diameter variations. Local axonal diameters were quantified at 150 nm intervals and defined as the equivalent diameter - the diameter of a circle with the same area as the axonal cross-section in the plane perpendicular to the local trajectory, as in^31^. The XNH image acquisition, segmentation and analysis is fully described in^29^. To produce triangulated surface meshes for simulations, the binary segmentations were first meshed using the isosurface() function in Matlab R2020, and then processed with the Corrective Smooth Modifier in Blender (RRID:SCR_008606) for reduction of high-frequency changes and irregularities. The meshes were decimated in Blender to reduce triangle density, while preserving the mesh volume, to reduce the computational burden of the simulations.

For these simulations, the main direction of each axon was calculated using a principal-component analysis of its trajectory, after which it could be rotated into the z-axis. 29 axons were then selected based on an upper limit of the volume of their bounding boxes; in the interest of feasibility of computing the corresponding internal gradients (described in 4.2.3). These make up the C5 substrates. Axonal configurations C1-C4 were first modelled as consisting of z-aligned cylindrical segments, after which one or more of the following properties from the C5 axons was inherited by deforming the cylindrical segments in the appropriate position along the axonal trajectory: mean axon diameter, longitudinal diameter variation, tortuous trajectories. Importantly, although the axonal cross-sections in the plane perpendicular to the local trajectory could be non-circular and non-symmetrical for the C5 configuration, this property was not inherited by C1-C4 for which the axonal cross-sections were circular.

To compute the susceptibility-induced internal gradients, myelin compartments were constructed artificially by expanding the axon mesh. The expansion was performed by translating each vertex of the mesh a distance $width_{myelin} = mean(r_{axon}) / g$, where $mean(r_{axon})$ is the mean radius of a given axon, and $g = 0.7$ is the g-ratio, in the direction away from the centreline of the original mesh. Each new mesh was then re-meshed, re-triangulated, and decimated in Blender (RRID:SCR_008606) to ensure the quality of the mesh-triangulation.

#### Hexagonally packed helical axons (intra- and extra-axonal compartment)

**Fig. 5:**
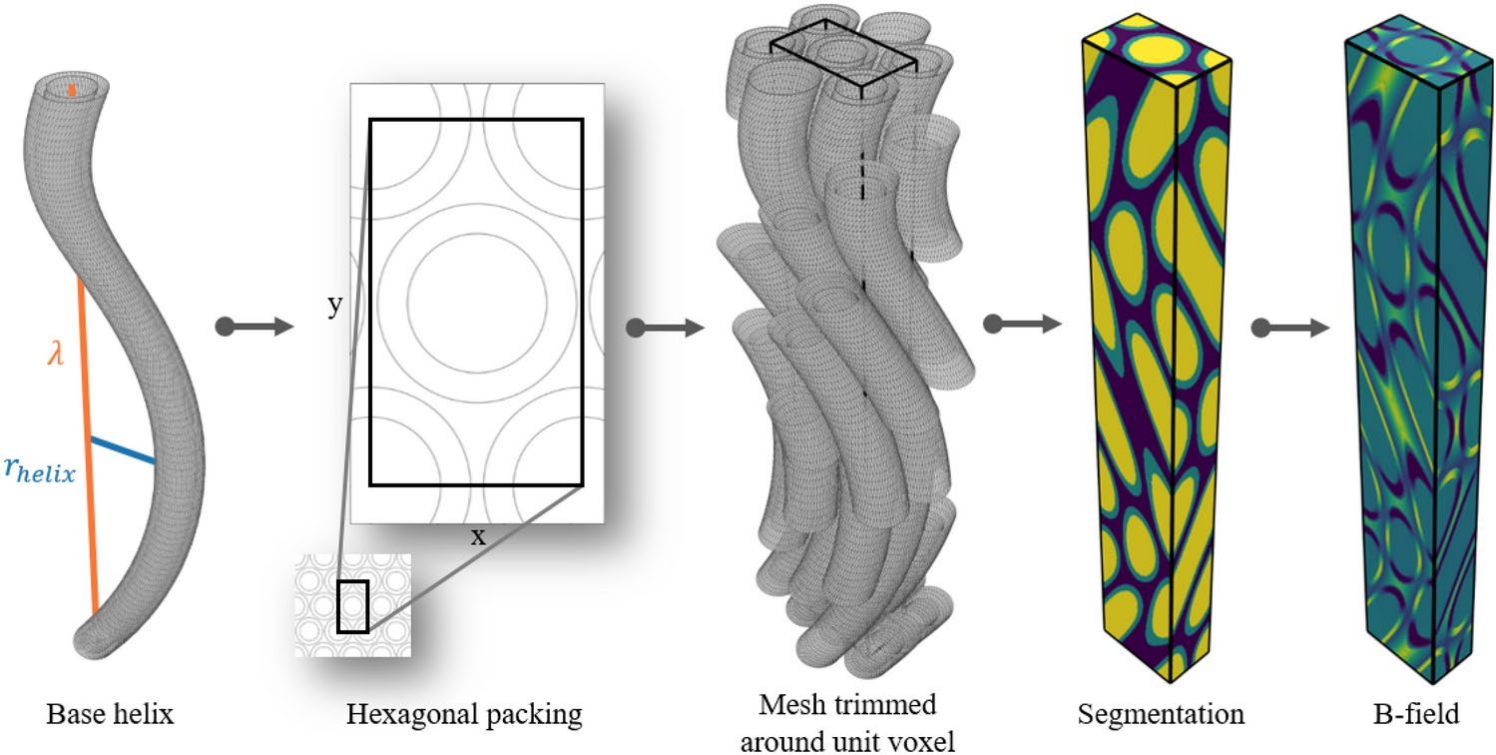
Pipeline for generating hexagonally packed helical axon substrates. A base axon is generated as a surface mesh, and defined by an axon radius $r_{axon}$, a g-ratio, a helix wavelength $\lambda$ (along $\hat{\textbf{z}}$), and a helix radius $r_{helix}$. The hexagonal packing is defined by an extra-axonal volume fraction (EAVF), and $r_{axon}$. Copies of the base axon are arranged according to the hexagonal packing, and the meshes are trimmed around the unit voxel of the hexagonal packing for computational efficiency. A compartment-wise segmentation is obtained as a voxel-representation of the surface mesh (intra-axonal in yellow, myelin in green, and extra-axonal in blue). B-fields are then computed by assigning magnetic susceptibilities to each compartment of the segmentation, and solving for $\textbf{B}_{\textbf{0}}$ applied in different directions $\text {angle}(\textbf{B}_{\textbf{0}}, \hat{\textbf{z}})$.

To study how the susceptibility-induced internal gradients of tortuous axons affect the signal in the intra-axonal and extra-axonal compartments relative to each other, we generated numerical mesh substrates of densely packed axons (Figs. 5 and 3a). Packing and undulation was designed with periodic boundary conditions for optimal memory usage during computation of the internal gradients (described in 4.2.3).

The myelinated axons (coaxial cylinders) are organized with hexagonal packing in the xy-plane (cross-sectional to the axons), and helical undulation along the z-axis (i.e. along the primary axon axis). The hexagonal packing and helical undulation impose translation-symmetry in all dimensions and enables the substrate to be fully described from a volume containing a single period along each dimension. We define this volume as the unit-voxel. The side lengths $(l_x, l_y, l_z)$ of the unit-voxel are given by

1 $$\begin{aligned} l_x= & \sqrt{ \frac{\pi \sqrt{3}(2 r_{myelin})^2}{6 (1 - EAVF)} }, \end{aligned}$$

2 $$\begin{aligned} l_y= & 2 \sqrt{(l_x)^2 - \left( \frac{l_x}{2}\right) ^2}, \end{aligned}$$

3 $$\begin{aligned} l_z= & \lambda , \end{aligned}$$

where $r_{myelin}$ is the outer radii of the myelinated axons in the xy-plane, *EAVF* is the extra-axonal volume fraction, and $\lambda$ is the wavelength of the undulation along the z-axis. Substrates were generated with extra-axonal volume fraction $EAVF = 0.2$ in accordance with^62^.

Myelinated axons were modelled as co-axial tubes with an outer radius $r_{myelin} = 1.9\,\mu \hbox {m}$ representing the radius of the myelin, an inner radius $r_{axon}=r_{myelin} \cdot g = 1.3\,\mu \hbox {m}$ representing the radius of the axon, where $g = 0.7$ is the g-ratio (as in other studies^63,64^). Axonal trajectory undulation is modelled as a helical trajectory with wavelength $\lambda$ and radius $r_{helix}$.

A surface mesh-representation of the axons was composed by stacking ring modules. The undulation causes axons to cross in and out of the unit-voxel. To save computation time during segmentation (described in 4.2.3) and Monte Carlo diffusion simulations (described in 4.2.4), the meshes are cropped module-wise around the unit-voxel.

#### Computation of susceptibility-induced internal gradients

To compute the susceptibility-induced internal gradients of the numerical substrates, we computed how the 3D distribution of magnetic susceptibilities (based on the three tissue compartments) perturbs an externally applied magnetic field (B-field). This is infeasible to do analytically for all but simple and analytically described geometries. To solve B-fields for the complex geometries presented here, we therefore implemented a finite-difference (FD) method.

The FD method requires a voxel-representation of the substrate, i.e. the volume of the unit-voxel partitioned into voxels. This was obtained with a ray-casting-based^65^ in-house GPU-compatible method. From the surface mesh-representation, the method segments the volume within the unit-voxel into three compartments (intra-axonal, extra-axonal, and myelin). All voxels in the myelin compartment of the voxel-representation are strictly contained within the myelin compartment of the surface mesh-representation; i.e. no myelin-voxels are transcending the meshes. This is crucial for preventing the B-field in the myelin compartment from contaminating the particles diffusing in the intra- and extra-axonal compartments. The resolution of the segmentations is a trade-off between descriptive accuracy of the morphology, and memory usage when computing the B-fields. We applied a desired resolution of $res_{desired} = 0.0655\,\mu \hbox {m}$ corresponding to 0.0252 times the mean diameter of the XNH-informed axon configurations and 0.0492 times the diameter of the helical axons. This resolution had to be slightly modified ($\pm 0.0001\,\mu \hbox {m}$) to match the unit-voxel of the individual substrate.

Exact susceptibility values for myelin, intra-axonal and extra-axonal compartments are unknown. We approximate the susceptibility of the intra- and extra-axonal compartments to that of water, i.e. $\chi _{water} = \chi _{intra} = \chi _{extra} = -9.04\cdot 10^{-6}$^52,53^, and the susceptibility of the myelin compartment to that of fat, i.e. $\chi _{fat} = -7.79\cdot 10^{-6}$^66^with a myelin water fraction (MWF) of 0.15 in accordance with findings for MWFs for normal-appearing white matter (NAWM) in^67,68^ by computing a weighted sum of the individual values, i.e. $\chi _{myelin} = MWF\cdot \chi _{water} + (1 - MWF)\cdot \chi _{fat} = -7.98\cdot 10^{-6}$.

The implementation of the FD method is inspired by Bhagwandien et al^26,33^, and applied to iteratively solve the magnetic scalar potential $\varvec{\Phi }$ from

4 $$\begin{aligned} \nabla \cdot (\varvec{\mu _r} \nabla (\varvec{\Phi })) = 0 \end{aligned}$$

where $\varvec{\mu _r} = \varvec{\chi } + 1$ is the volumetric distribution of magnetic permeability of the substrate relative to that of vacuum. From $\varvec{\Phi }$ the susceptibility-induced permutation of the magnetic field $\Delta \textbf{B}$ is then computed by

5 $$\begin{aligned} \Delta \textbf{B} = \mu _0 \varvec{\mu _r}\left( 1-\frac{2}{3}(\varvec{\mu _r}-1)\right) (-\nabla \varvec{\Phi }) \end{aligned}$$

where $\mu _0$ is the magnetic permeability of vacuum. B-fields were computed for $B_0 = 7\,\,\hbox {T}$ at different orientations $\text {angle}(\textbf{B}_{\textbf{0}},\hat{\textbf{z}}) = (0, 15, 30, 45, 60, 75, 90)$ deg between the primary orientation axis of the axons $\hat{\textbf{z}}$ and $\textbf{B}_{\textbf{0}}$.

Due to the anisotropic organization of the lipids within the myelin, the B-field perturbations can be divided into an isotropic contribution and an anisotropic contribution^27^, where the isotropic contribution comes from the bulk of the myelin, while the anisotropic contribution comes from the dipole effects of the lipids. Here, only the isotropic contribution is considered.

#### Monte Carlo diffusion simulations and integration of internal gradients

To generate dMRI signals from the numerical substrates, we first performed Monte Carlo diffusion simulations to obtain the diffusion trajectories of particles restricted by the surface mesh-representations of the substrates. Then, we computed the resulting dMRI signals taking into account both the (diffusion encoding) external gradients and the (susceptibility-induced) internal gradients.

The diffusion trajectories were obtained for the surface mesh-representations of the numerical substrates with the MC/DC Simulator^69^ with diffusion coefficient $D_0 = 0.6\cdot 10^{-6}\,\hbox {mm}^2/\hbox {ms}$(as applied for ex vivo elsewhere^45^), step length $l_{step} = 0.11\,\mu \hbox {m}$, and particle density $\rho _{particles} = 15 \,\mu \text {m}^{-3}$ (corresponding to between 7360 and 23271 intra-axonal particles per XNH-informed substrate, and 14177 extra-axonal and 27786 intra-axonal per hexagonally packed substrate). Signal from the myelin compartment was neglected due to its short T2 ($\sim 20\,\hbox {ms}$) and T2* ($\sim 10\,\hbox {ms}$)^70,71^ compared to the applied TE. Particle initialisation was done only in the intra- and extra-axonal compartments, and permeability was set to zero.

For the XNH-informed axon configurations, particle initialisation was only done in the intra-axonal compartment. Particles were initialised at a minimum distance of $20\, \mu \hbox {m}$ from the closed ends to avoid probing these.

The simulation experiment used the same acquisition setup (b-values, b-vectors, TE) as in the ex vivo experiment (described in 4.3.2). The experiment was repeated at different orientations w.r.t. $\textbf{B}_{\textbf{0}}$ at $\text {angle}(\textbf{B}_{\textbf{0}},\hat{\textbf{z}}) = (0, 15, 30, 45, 60, 75, 90)$ deg.

The dMRI signal was computed from the simulated diffusion trajectories by accumulating both the phase contribution from the external gradients of the PGSE sequence, and the phase contribution from the internal gradients caused at a given $\theta _z$. The signal is given by^72^

6 $$\begin{aligned} S_{eff} = \sum _{j=1}^{N_{particles}} \cos \left( \phi _{ext,j} + \phi _{int,j}\right) \end{aligned}$$

where $\phi$ is the temporally accumulated phase of a particle computed by

7 $$\begin{aligned} \phi _{ext,j}= & \gamma \left( \sum _{t=t_1}^{t_2}\mathbf {g_{ext}}\cdot \mathbf {x_{j}}(t) - \sum _{t=t_3}^{t_4}\mathbf {g_{ext}}\cdot \mathbf {x_{j}}(t) \right) \end{aligned}$$

8 $$\begin{aligned} \phi _{int,j}= & \gamma \left( \sum _{t=0}^{\tau }\Delta \textbf{B}(\mathbf {x_j}(t)) -\sum _{t=\tau }^{TE}\Delta \textbf{B}(\mathbf {x_j}(t)) \right) \end{aligned}$$

where $\mathbf {g_{ext}}$ is the external gradient field, $\Delta \textbf{B}$ is the susceptibility-induced permutation of the magnetic field characterising the internal gradients, $\tau =TE/2$ is the inversion time, *t* is time, and $t_1$, $t_2 = t_1 + \delta$, $t_3 = t_1 + \Delta$, and $t_4 = t_3 + \delta$ are the temporal boundaries of the external gradients, and $\delta$ is the duration of the external gradients.

### Ex vivo MRI experiments

A tissue block of $(\sim 17\,\hbox {mm})^3$ was dissected from a whole perfusion fixated vervet monkey brain, and scanned at different orientations w.r.t. $\textbf{B}_{\textbf{0}}$ to validate the orientation-dependences observed for the numerical substrates.

#### Rotation device

**Fig. 6:**
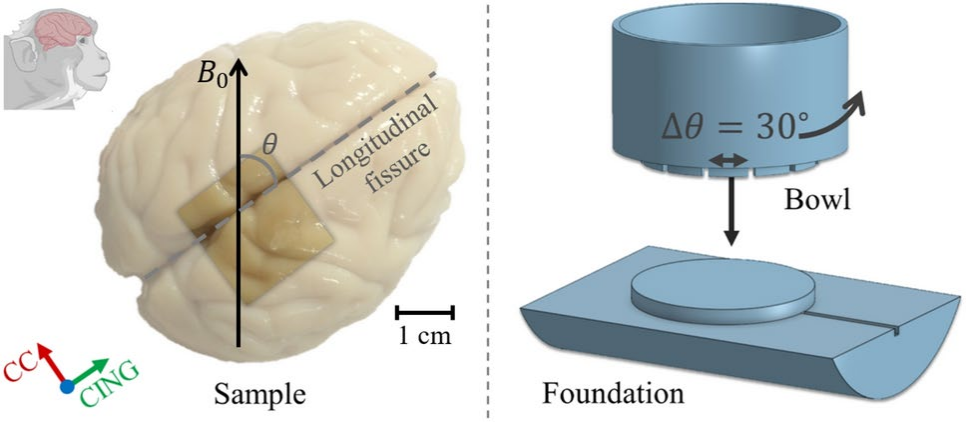
Ex vivo experimental set-up. **a** Indication of the dissected tissue block, and how the orientation of the tissue w.r.t. $\textbf{B}_{\textbf{0}}$ was defined based on the longitudinal fissure. **b** Rotation device tailored for the RF-coil. Some figure elements in a) were created with BioRender.com.

The tissue block was placed in a rotation device as shown in Fig. 6. The rotation device was tailored for the RF-coil, such that the centre of the bowl is the centre of the coil when placed on the associated foundation. The sides of the bowl has the geometry of a hollow coaxial cylinder, which in the ideal case induces no internal gradients inside the bowl^26^. Orientation of the bowl was controlled with 30 deg increments according to the foot of the bowl. The tissue sample was moulded in 2% PBS-agar^73^ to stabilize it mechanically in the bowl. The bowl was sealed off using Parafilm®and nitrile rubber to avoid dehydration during scanning. The bowl was placed on the foundation to control the rotation. The orientation of the brain w.r.t. $\textbf{B}_{\textbf{0}}$ is measured as the angle between $\textbf{B}_{\textbf{0}}$ and the longitudinal fissure.

#### MRI scan parameters

Two scan-series were acquired. Within each series, the scan-protocol (described below) was repeated for different orientations w.r.t. $\textbf{B}_{\textbf{0}}$ at $\theta = (0, 90, 30, 60)$ deg (Fig. 6). The order was pseudorandomized in order to exclude temporal bias in the analysis. Field-of-view was aligned with the longitudinal fissure at each scan.

Scans were performed on a 7 T Bruker Biospec 70/20 preclinical MRI scanner with maximum gradient strength of 600 mT/m, and a 1H transmit-receive volume quadrature RF-coil with inner diameter of 40 mm. Imaging was obtained with a 2D PGSE sequence with slice direction along z, $300 \,\mu \hbox {m}$ isotropic resolution, acquired with 38 slices and a matrix-size of 170x87, single line read-out, bandwidth = 18.5 kHz, TE/TR = 36/3200 ms. PGSE parameters were $\delta = 7.2\,\hbox {ms}$, $\Delta = 20.2\,\hbox {ms}$, varying gradient strength, and $b = (50, 1000, 3000, 4000)\,\hbox {s/mm}^2$ and $b = (50, 1000, 3000, 4000, 8000, 12000, 20000)\,\hbox {s/mm}^2$ for scan-series 1 and 2 respectively. With a minimum b-value of $50\,\hbox {s/mm}^2$ the need of signal-correcting crusher gradients was avoided. While crusher gradients would contribute with additional cross-terms in a fixed direction, a b-value of $50\,\hbox {s/mm}^2$is negligible and provides cross-terms under same conditions as for the larger b-values. Diffusion weighting was repeated with 21 uniformly distributed gradient vectors over a half-sphere. The directional scheme was repeated with opposite polarity for later correction of cross-terms between external diffusion gradients, image slice gradients, and macroscopic internal gradients^20^. The effect on the cross-terms between the external diffusion gradients and image slice gradients is shown in Fig. S7. Across all scans, the SNR in CC at $b = 50\,\hbox {s/mm}^2$ was $163\pm 11$ before noise suppression and $701\pm 127$ after. Across the scans of scan-series 2, the SNR in CC at $b = 20000\,\hbox {s/mm}^2$ was $57\pm 11$ prior to noise suppression and $206\pm 21$ after.

Prior to the scan session the rotation setup with the agar embedded tissue was placed in the RF coil at room temperature at least 5 hours prior to scanning for temperature stabilization. To reduce short-term instabilities (i.e. temperature variations in the magnet and motion effect due to setup handling and initial heating of the gradient hardware) a dummy dMRI scan of $>2$ hours was collected before the actual DWI scans. To reduce temperature variation of the tissue during scanning a constant airflow of room temperature was ensured around the sample.

#### Image post-processing

First, Gibbs ringing effects and noise were suppressed for all dMRI scans using gibbs_removal() and mppca()from the DIPY library^74^. Then, all scans were co-registered to a slightly rotated version of one of the scans using aling()from the DIPY library. To minimize cross-term effects between imaging gradients, macroscopic background field inhomogeneities and the diffusion gradients cross-term correction^20^ was applied (Fig. S7).

### Analysis

#### Diffusion tensor modelling (in silico and ex vivo)

To assess the anisotropy of the apparent diffusion, we fitted the widely applied diffusion tensor (DT) model^36,37^. Although the model is not capable of fully characterising the non-Gaussian diffusion in white matter, it does capture the principal anisotropy of the signal. Changes in this principal signal anisotropy are quantified to estimate the dependence of the orientation between axons and $\textbf{B}_{\textbf{0}}$. The DT model was fitted with the DIPY library^74^ to the intervals $b = (50, 1000, 3000, 4000)\,\hbox {s/mm}^2$, $b = (4000, 8000, 12000)\,\hbox {s/mm}^2$, and $b = (8000, 12000, 20000)\,\hbox {s/mm}^2$. The mean diffusivity (MD, mean of all eigenvalues), the axial diffusivity (AD, first eigenvalue), the radial diffusivity (RD, mean of second and third eigenvalue), and the first eigenvector ($\textbf{e}_{\textbf{1}}$) were extracted for analysis.

In silico, the intra-axonal orientation-dependence trends (Fig. 1) were categorized by quantifying minimum and maximum deviations between the cases with and without the integration of internal gradients. Ex vivo, orientation-dependence was then quantified based on the categories observed in silico by fitting each of the phenomenological models

A. $f(x) = p_0$ (no orientation-dependence)
B. $f(x) = p_0 + p_1 \cdot \cos (x)^2$ (largest effect at $\text {angle}(\textbf{B}_{\textbf{0}}, \hat{\textbf{z}}) = 90$ deg)
C. $f(x) = p_0 + p_1 \cdot \cos (2x)^2$ (largest effect at $\text {angle}(\textbf{B}_{\textbf{0}}, \hat{\textbf{z}}) = 45$ deg)
D. $f(x) = p_0 + p_1 \cdot \cos (x - \pi /2)^2$ (largest effect at $\text {angle}(\textbf{B}_{\textbf{0}}, \hat{\textbf{z}}) = 0$ deg)

where $p_0$ is a constant off-set, and $p_1\ge 0$ represents the degree of orientation-dependence. The relative performances of the models are ranked by their respective Akaike information criterion (AIC)^75,76^

9 $$\begin{aligned} AIC = 2K + N\cdot \ln \left( \frac{\text {RSS}}{N}\right) , \end{aligned}$$

where *K* is the number of fitting parameters, *N* is the number of data points, and *RSS* is the residual sum of squares. To simplify the interpretation, the AIC-values were rescaled as

10 $$\begin{aligned} \Delta AIC = AIC - AIC_{min}, \end{aligned}$$

where $AIC_{min}$ is the minimum AIC obtained for any of the models. $\Delta AIC$ is the information loss experienced by fitting the given model rather than the best model. As suggested in^76^ the relative support of the models is assessed according to the following scheme: $\Delta AIC \le 2$ indicates substantial support, $4 \le \Delta AIC \le 7$ indicates considerably less support, and $\Delta AIC> 10$ indicates essentially no support.

#### Regions of interest from ex vivo images

We focus the analysis on the body of the corpus callosum (CC) and the mid-cingula (CING-L and CING-R). These bundles are oriented in the $\hat{\textbf{x}}$ and $\hat{\textbf{y}}$ directions of the field-of-view, respectively. With $\hat{\textbf{z}}$ as the axis of rotation, CC will be parallel with $\textbf{B}_{\textbf{0}}$ when CING-L and CING-R are orthogonal with $\textbf{B}_{\textbf{0}}$, and vice versa.

Voxels of interest were extracted based on manual segmentation in FSLeyes^77^ of the volume which was kept static during the co-registration, and additional thresholding of the individual volumes. The thresholding criteria were: $\hbox {FA}> 0.6$, and angle with respect to the estimated fiber-direction of interest $< 30$ degrees. For scan series 1, this results in $156\pm 0$, $149\pm 16$, and $96\pm 15$ voxels for CC, CING-L and CING-R, respectively. For scan series 2, it results in $318\pm 12$, $234\pm 31$, and $159\pm 31$ voxels for CC, CING-L and CING-R, respectively.

## Supplementary Information

Supplementary Information.

## Data Availability

The datasets generated and analysed during this study is available at www.drcmr.dk/susceptibility-and-axon-morphology-dataset. The code to reproduce the analyses and numerical experiments is available at www.github.com/MaP-science/susceptibility-and-axon-morphology.

## References

[1] Ogawa, S, Lee, T. M., Kay, A. R. & Tank, D. W. Brain magnetic resonance imaging with contrast dependent on blood oxygenation. *Proc. Natl. Acad. Sci. U. S. A.* **87**, 9868–9872 (1990). 10.1073/pnas.87.24.9868 PMC55275 2124706

[2] Haacke, E. M., Xu, Y., Cheng, Y.-C.N. & Reichenbach, J. R. Susceptibility weighted imaging (SWI). *Magn. Reson. Med.* **52**, 612–618 (2004). 15334582 10.1002/mrm.20198

[3] Laule, C. et al. Magnetic resonance imaging of myelin. *Neurotherapeutics* **4**, 460–484 (2007). 17599712 10.1016/j.nurt.2007.05.004 PMC7479725

[4] Nunes, D., Cruz, T. L., Jespersen, S. N. & Shemesh, N. Mapping axonal density and average diameter using non-monotonic time-dependent gradient-echo MRI. *J. Magn. Reson.* **277**, 117–130 (2017). 28282586 10.1016/j.jmr.2017.02.017

[5] He, X. & Yablonskiy, D. A. Biophysical mechanisms of phase contrast in gradient echo MRI. *Proc. Natl. Acad. Sci. U. S. A.* **106**, 13558–13563 (2009). 19628691 10.1073/pnas.0904899106 PMC2714760

[6] Liu, C. Susceptibility tensor imaging. *Magn. Reson. Med.* **63**, 1471–1477 (2010). 20512849 10.1002/mrm.22482 PMC2990786

[7] Álvarez, G. A., Shemesh, N. & Frydman, L. Internal gradient distributions: A susceptibility-derived tensor delivering morphologies by magnetic resonance. *Sci. Rep.* **7**, 3311 (2017). 28607445 10.1038/s41598-017-03277-9 PMC5468317

[8] Caporale, A. et al. The γ-parameter of anomalous diffusion quantified in human brain by MRI depends on local magnetic susceptibility differences. *Neuroimage* **147**, 619–631 (2017). 28011255 10.1016/j.neuroimage.2016.12.051

[9] Sandgaard, A. D., Shemesh, N., Kiselev, V. G. & Jespersen, S. N. Larmor frequency shift from magnetized cylinders with arbitrary orientation distribution. *NMR Biomed.* **36**, e4859 (2023). 36285793 10.1002/nbm.4859 PMC10078263

[10] Beaulieu, C. & Allen, P. S. An in vitro evaluation of the effects of local magnetic-susceptibility-induced gradients on anisotropic water diffusion in nerve. *Magn. Reson. Med.* **36**, 39–44 (1996). 8795018 10.1002/mrm.1910360108

[11] Trudeau, J. D., Dixon, W. T. & Hawkins, J. The effect of inhomogeneous sample susceptibility on measured diffusion anisotropy using NMR imaging. *J. Magn. Reson. B* **108**, 22–30 (1995). 7627433 10.1006/jmrb.1995.1098

[12] Clark, C. A., Barker, G. J. & Tofts, P. S. An in vivo evaluation of the effects of local magnetic susceptibility-induced gradients on water diffusion measurements in human brain. *J. Magn. Reson.* **141**, 52–61 (1999). 10527743 10.1006/jmre.1999.1872

[13] Kleban, E., Jones, D. K. & Tax, C. M. W. The impact of head orientation with respect to B0 on diffusion tensor MRI measures. *Imaging Neuroscience* **1**, 1–17 (2023). 38405373 10.1162/imag_a_00012 PMC10884544

[14] Novikov, D. S., Reisert, M. & Kiselev, V. G. Effects of mesoscopic susceptibility and transverse relaxation on diffusion NMR. *J. Magn. Reson.* **293**, 134–144 (2018). 30012279 10.1016/j.jmr.2018.06.007

[15] Knight, M. J., Dillon, S., Jarutyte, L. & Kauppinen, R. A. Magnetic Resonance Relaxation Anisotropy: Physical Principles and Uses in Microstructure Imaging. *Biophys. J.* **112**, 1517–1528 (2017). 28402893 10.1016/j.bpj.2017.02.026 PMC5390049

[16] Stejskal, E. O. & Tanner, J. E. Spin diffusion measurements: Spin echoes in the presence of a time-dependent field gradient. *J. Chem. Phys.* **42**, 288–292 (1965).

[17] Hahn, E. L. Spin Echoes. *Phys. Rev.* **80**, 580–594 (1950).

[18] Szczepankiewicz, F. & Sjölund, J. Cross-term-compensated gradient waveform design for tensor-valued diffusion MRI. *J. Magn. Reson.* **328**, 106991 (2021). 33984713 10.1016/j.jmr.2021.106991

[19] Neeman, M, Freyer, J. P. & Sillerud, L. O. A simple method for obtaining cross-term-free images for diffusion anisotropy studies in NMR microimaging. *Magn. Reson. Med.* **21**, 138–143 (1991). 10.1002/mrm.1910210117 1943671

[20] Jara, H. & Wehrli, F. W. Determination of background gradients with diffusion MR imaging. *J. Magn. Reson. Imaging* **4**, 787–797 (1994). 7865938 10.1002/jmri.1880040608

[21] Hong, X. & Thomas Dixon, W. Measuring diffusion in inhomogeneous systems in imaging mode using antisymmetric sensitizing gradients. *J. Magn. Reson.* **99**, 561–570 (1992).

[22] Galvosas, P., Stallmach, F. & Kärger, J. Background gradient suppression in stimulated echo NMR diffusion studies using magic pulsed field gradient ratios. *J. Magn. Reson.* **166**, 164–173 (2004). 14729028 10.1016/j.jmr.2003.09.012

[23] Li, W., Wu, B., Avram, A. V. & Liu, C. Magnetic susceptibility anisotropy of human brain in vivo and its molecular underpinnings. *Neuroimage* **59**, 2088–2097 (2012). 22036681 10.1016/j.neuroimage.2011.10.038 PMC3254777

[24] Oh, S.-H., Kim, Y.-B., Cho, Z.-H. & Lee, J. Origin of B0 orientation dependent R2(*) (=1/T2(*)) in white matter. *Neuroimage* **73**, 71–79 (2013). 23376494 10.1016/j.neuroimage.2013.01.051 PMC3638902

[25] Novikov, D. S., Fieremans, E., Jespersen, S. N. & Kiselev, V. G. Quantifying brain microstructure with diffusion MRI: Theory and parameter estimation. *NMR Biomed.* **32**, e3998 (2019). 30321478 10.1002/nbm.3998 PMC6481929

[26] Bhagwandien, R., Moerland, M. A., Bakker, C. J., Beersma, R. & Lagendijk, J. J. Numerical analysis of the magnetic field for arbitrary magnetic susceptibility distributions in 3D. *Magn. Reson. Imaging* **12**, 101–107 (1994). 8295497 10.1016/0730-725x(94)92357-4

[27] Wharton, S. & Bowtell, R. Fiber orientation-dependent white matter contrast in gradient echo MRI. *Proc. Natl. Acad. Sci. U. S. A.* **109**, 18559–18564 (2012). 23091011 10.1073/pnas.1211075109 PMC3494918

[28] Sukstanskii, A. L. & Yablonskiy, D. A. On the role of neuronal magnetic susceptibility and structure symmetry on gradient echo MR signal formation. *Magn. Reson. Med.* **71**, 345–353 (2014). 23382087 10.1002/mrm.24629 PMC3657601

[29] Andersson, M. et al. Axon morphology is modulated by the local environment and impacts the noninvasive investigation of its structure-function relationship. *Proceedings of the National Academy of Sciences* **117**, 33649–33659 (2020). 10.1073/pnas.2012533117 PMC7777205 33376224

[30] Lee, H.-H. et al. Along-axon diameter variation and axonal orientation dispersion revealed with 3D electron microscopy: implications for quantifying brain white matter microstructure with histology and diffusion MRI. *Brain Struct. Funct.* **224**, 1469–1488 (2019). 30790073 10.1007/s00429-019-01844-6 PMC6510616

[31] Abdollahzadeh, A., Belevich, I., Jokitalo, E., Tohka, J. & Sierra, A. Automated 3D Axonal Morphometry of White Matter. *Sci. Rep.* **9**, 6084 (2019). 30988411 10.1038/s41598-019-42648-2 PMC6465365

[32] Xu, T., Foxley, S., Kleinnijenhuis, M., Chen, W. C. & Miller, K. L. The effect of realistic geometries on the susceptibility-weighted MR signal in white matter. *Magn. Reson. Med.* **79**, 489–500 (2018). 28394030 10.1002/mrm.26689 PMC6585669

[33] Bhagwandien, R. et al. Numerical analysis of the magnetic field for arbitrary magnetic susceptibility distributions in 2D. *Magn. Reson. Imaging* **10**, 299–313 (1992). 1564997 10.1016/0730-725x(92)90489-m

[34] Palombo, M., Gentili, S., Bozzali, M., Macaluso, E. & Capuani, S. New insight into the contrast in diffusional kurtosis images: does it depend on magnetic susceptibility?. *Magn. Reson. Med.* **73**, 2015–2024 (2015). 24894844 10.1002/mrm.25308

[35] Tax, C. M. W. et al. Measuring compartmental T2-orientational dependence in human brain white matter using a tiltable RF coil and diffusion-T2 correlation MRI. *Neuroimage* **236**, 117967 (2021). 33845062 10.1016/j.neuroimage.2021.117967 PMC8270891

[36] Basser, P. J., Mattiello, J. & LeBihan, D. MR diffusion tensor spectroscopy and imaging. *Biophys. J.* **66**, 259–267 (1994). 8130344 10.1016/S0006-3495(94)80775-1 PMC1275686

[37] Soares, J. M., Marques, P., Alves, V. & Sousa, N. A hitchhiker’s guide to diffusion tensor imaging. *Front. Neurosci.* **7**, 31 (2013). 23486659 10.3389/fnins.2013.00031 PMC3594764

[38] Trapp, B. D. et al. Axonal transection in the lesions of multiple sclerosis. *N. Engl. J. Med.* **338**, 278–285 (1998). 9445407 10.1056/NEJM199801293380502

[39] Langford, T. D. et al. Severe, demyelinating leukoencephalopathy in AIDS patients on antiretroviral therapy. *AIDS* **16**, 1019–1029 (2002). 11953468 10.1097/00002030-200205030-00008 PMC3548569

[40] Corral, I. et al. Focal monophasic demyelinating leukoencephalopathy in advanced HIV infection. *Eur. Neurol.* **52**, 36–41 (2004). 15237251 10.1159/000079416

[41] Sormani, M. P., Arnold, D. L. & De Stefano, N. Treatment effect on brain atrophy correlates with treatment effect on disability in multiple sclerosis. *Ann. Neurol.* **75**, 43–49 (2014). 24006277 10.1002/ana.24018

[42] Wattjes, M. P. et al. Evidence-based guidelines: MAGNIMS consensus guidelines on the use of MRI in multiple sclerosis-establishing disease prognosis and monitoring patients. *Nat. Rev. Neurol.* **11**, 597–606 (2015). 26369511 10.1038/nrneurol.2015.157

[43] Garthwaite, G, Brown, G, Batchelor, A. M., Goodwin, D. A. & Garthwaite, J. Mechanisms of ischaemic damage to central white matter axons: a quantitative histological analysis using rat optic nerve. *Neuroscience* **94**, 1219–1230 (1999). 10.1016/s0306-4522(99)00389-9 10625062

[44] Tang-Schomer, M. D., Johnson, V. E., Baas, P. W., Stewart, W. & Smith, D. H. Partial interruption of axonal transport due to microtubule breakage accounts for the formation of periodic varicosities after traumatic axonal injury. *Exp. Neurol.* **233**, 364–372 (2012). 22079153 10.1016/j.expneurol.2011.10.030 PMC3979336

[45] Alexander, D. C. et al. Orientationally invariant indices of axon diameter and density from diffusion MRI. *Neuroimage* **52**, 1374–1389 (2010). 20580932 10.1016/j.neuroimage.2010.05.043

[46] Fan, Q. et al. Axon diameter index estimation independent of fiber orientation distribution using high-gradient diffusion MRI. *Neuroimage* **222**, 117197 (2020). 32745680 10.1016/j.neuroimage.2020.117197 PMC7736138

[47] Zhang, H., Schneider, T., Wheeler-Kingshott, C. A. & Alexander, D. C. NODDI: practical in vivo neurite orientation dispersion and density imaging of the human brain. *Neuroimage* **61**, 1000–1016 (2012). 22484410 10.1016/j.neuroimage.2012.03.072

[48] Andersson, M. et al. Does powder averaging remove dispersion bias in diffusion MRI diameter estimates within real 3D axonal architectures?. *Neuroimage* **248**, 118718 (2022). 34767939 10.1016/j.neuroimage.2021.118718

[49] Nilsson, M., Lätt, J., Ståhlberg, F., van Westen, D. & Hagslätt, H. The importance of axonal undulation in diffusion MR measurements: a Monte Carlo simulation study. *NMR Biomed.* **25**, 795–805 (2012). 22020832 10.1002/nbm.1795

[50] Lee, H.-H., Jespersen, S. N., Fieremans, E. & Novikov, D. S. The impact of realistic axonal shape on axon diameter estimation using diffusion MRI. *Neuroimage* **223**, 117228 (2020). 32798676 10.1016/j.neuroimage.2020.117228 PMC7806404

[51] Chen, W. C., Foxley, S. & Miller, K. L. Detecting microstructural properties of white matter based on compartmentalization of magnetic susceptibility. *Neuroimage* **70**, 1–9 (2013). 23268785 10.1016/j.neuroimage.2012.12.032 PMC3591254

[52] Hopkins, J. A. & Wehrli, F. W. Magnetic susceptibility measurement of insoluble solids by NMR: magnetic susceptibility of bone. *Magn. Reson. Med.* **37**, 494–500 (1997). 9094070 10.1002/mrm.1910370404

[53] Collins, C. M., Yang, B., Yang, Q. X. & Smith, M. B. Numerical calculations of the static magnetic field in three-dimensional multi-tissue models of the human head. *Magn. Reson. Imaging* **20**, 413–424 (2002). 12206867 10.1016/s0730-725x(02)00507-6

[54] Kawamura, Y., Sakurai, I., Ikegami, A. & Iwayanagi, S. Magneto-Orientation of Phospholipids. *Mol. Cryst. Liq. Cryst.* **67**, 77–87 (1981).

[55] Nazemorroaya, A. *et al.* Developing formalin-based fixative agents for post mortem brain MRI at 9.4 T. *Magn. Reson. Med.* **87**, 2481–2494 (2022). 10.1002/mrm.29122 34931721

[56] Möbius, W., Nave, K.-A. & Werner, H. B. Electron microscopy of myelin: Structure preservation by high-pressure freezing. *Brain Res.* **1641**, 92–100 (2016). 26920467 10.1016/j.brainres.2016.02.027

[57] Chan, K.-S. et al. Imaging white matter microstructure with gradient-echo phase imaging: Is ex vivo imaging with formalin-fixed tissue a good approximation of the in vivo brain?. *Magn. Reson. Med.* **88**, 380–390 (2022). 35344591 10.1002/mrm.29213 PMC9314807

[58] D’Arceuil, H. E., Westmoreland, S. & de Crespigny, A. J. An approach to high resolution diffusion tensor imaging in fixed primate brain. *Neuroimage* **35**, 553–565 (2007). 17292630 10.1016/j.neuroimage.2006.12.028

[59] Birkl, C. et al. Effects of formalin fixation and temperature on MR relaxation times in the human brain. *NMR Biomed.* **29**, 458–465 (2016). 26835664 10.1002/nbm.3477

[60] Dyrby, T. B., Innocenti, G. M., Bech, M. & Lundell, H. Validation strategies for the interpretation of microstructure imaging using diffusion MRI. *Neuroimage* **182**, 62–79 (2018). 29920374 10.1016/j.neuroimage.2018.06.049

[61] Dyrby, T. B. et al. An ex vivo imaging pipeline for producing high-quality and high-resolution diffusion-weighted imaging datasets. *Hum. Brain Mapp.* **32**, 544–563 (2011). 20945352 10.1002/hbm.21043 PMC6870191

[62] Nicholson, C. & Syková, E. Extracellular space structure revealed by diffusion analysis. *Trends Neurosci.* **21**, 207–215 (1998). 9610885 10.1016/s0166-2236(98)01261-2

[63] Tomasi, S., Caminiti, R. & Innocenti, G. M. *Areal Differences in Diameter and Length of Corticofugal Projections* 2012. 10.1093/cercor/bhs011 22302056

[64] Caminiti, R. et al. Diameter, length, speed, and conduction delay of callosal axons in macaque monkeys and humans: comparing data from histology and magnetic resonance imaging diffusion tractography. *J. Neurosci.* **33**, 14501–14511 (2013). 24005301 10.1523/JNEUROSCI.0761-13.2013 PMC6618375

[65] Roth, S. D. Ray casting for modeling solids. *Computer Graphics and Image Processing* **18**, 109–144 (1982).

[66] Schenck, J. F. The role of magnetic susceptibility in magnetic resonance imaging: MRI magnetic compatibility of the first and second kinds. *Med. Phys.* **23**, 815–850 (1996). 8798169 10.1118/1.597854

[67] Faizy, T. D. et al. The Myelin Water Fraction Serves as a Marker for Age-Related Myelin Alterations in the Cerebral White Matter - A Multiparametric MRI Aging Study. *Front. Neurosci.* **14**, 136 (2020). 32153358 10.3389/fnins.2020.00136 PMC7050496

[68] Borich, M. R., Mackay, A. L., Vavasour, I. M., Rauscher, A. & Boyd, L. A. Evaluation of white matter myelin water fraction in chronic stroke. *Neuroimage Clin* **2**, 569–580 (2013). 24179808 10.1016/j.nicl.2013.04.006 PMC3777839

[69] Rafael-Patino, J. et al. Robust Monte-Carlo Simulations in Diffusion-MRI: Effect of the Substrate Complexity and Parameter Choice on the Reproducibility of Results. *Front. Neuroinform.* **14**, 8 (2020). 32210781 10.3389/fninf.2020.00008 PMC7076166

[70] Lee, J. et al. So You Want to Image Myelin Using MRI: An Overview and Practical Guide for Myelin Water Imaging. *J. Magn. Reson. Imaging* **53**, 360–373 (2021). 32009271 10.1002/jmri.27059

[71] Laule, C. et al. Myelin water imaging of multiple sclerosis at 7 T: correlations with histopathology. *Neuroimage* **40**, 1575–1580 (2008). 18321730 10.1016/j.neuroimage.2007.12.008

[72] Hall, M. G. & Alexander, D. C. Convergence and parameter choice for Monte-Carlo simulations of diffusion MRI. *IEEE Trans. Med. Imaging* **28**, 1354–1364 (2009). 19273001 10.1109/TMI.2009.2015756

[73] Rossi, F., Perale, G. & Masi, M. Biological buffered saline solution as solvent in agar-carbomer hydrogel synthesis. *Chem. Pap.* **64**, 573–578 (2010).

[74] Garyfallidis, E. et al. Dipy, a library for the analysis of diffusion MRI data. *Front. Neuroinform.* **8**, 8 (2014). 24600385 10.3389/fninf.2014.00008 PMC3931231

[75] Akaike, H. *in Springer Series in Statistics 215–222* (Springer, New York, New York, NY, 1974).

[76] Burnham, K. P. & Anderson, D. R. Multimodel Inference: Understanding AIC and BIC in Model Selection. *Sociol. Methods Res.* **33**, 261–304 (2004).

[77] McCarthy, P. *FSLeyes* 2019.

