# Supplementary material for: Susceptibility-induced internal gradients reveal axon morphology and cause anisotropic effects in the diffusion-weighted MRI signal
Source: Sci Rep. 2024 Nov 28;14:29636. doi: 10.1038/s41598-024-79043-5 (PMC11605075; doi:10.1038/s41598-024-79043-5)
Supplement: Supplementary file 1 — Supplementary Information. [file 41598_2024_79043_MOESM1_ESM.pdf]

1 Supplementary material

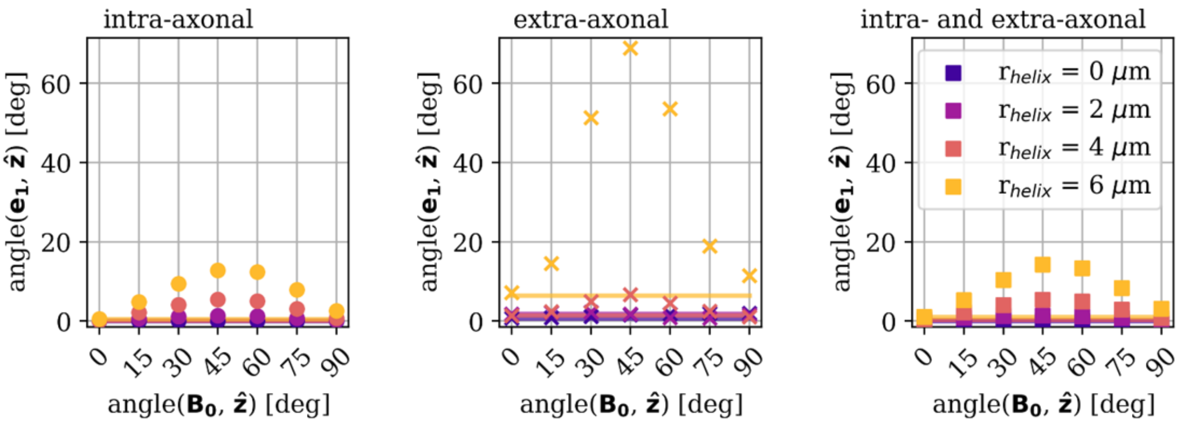

Figure S1: Susceptibility-induced internal fields affect the estimated fiber direction. We estimate the effect by computing the angle between the first eigenvector of the DT fit ($\mathbf{e_1}$) and the primary axis of the axons ($\hat{\mathbf{z}}$). Markers indicate the DT metrics fitted to $S_{eff}(b)$. Lines indicate the DT metrics fitted to $S_{des}(b)$.

It is seen that the angle is affected both by the helical radius of the axons r_{helix} (i.e. the degree of undulation), and by $angle(\hat{\mathbf{z}}, B_0)$ (i.e. orientation w.r.t. $\mathbf{B_0}$). When no internal gradients are taken into account (lines) there is a substantial deviation between $\hat{\mathbf{z}}$ and $\mathbf{e_1}$ for $r_{helix} \geq 3.0\mu$m, as expected for high degrees of undulation. When internal gradients are taken into account (markers) the deviation between $\mathbf{e_1}$ and $\hat{\mathbf{z}}$ becomes dependent on $angle(\hat{\mathbf{z}}, B_0)$ for $r_{helix} \geq 4.0\mu$m. This orientation-dependence means that the estimated ADs and RDs will not be in the true axial and radial directions of the substrates, but instead biased by the susceptibility effects.

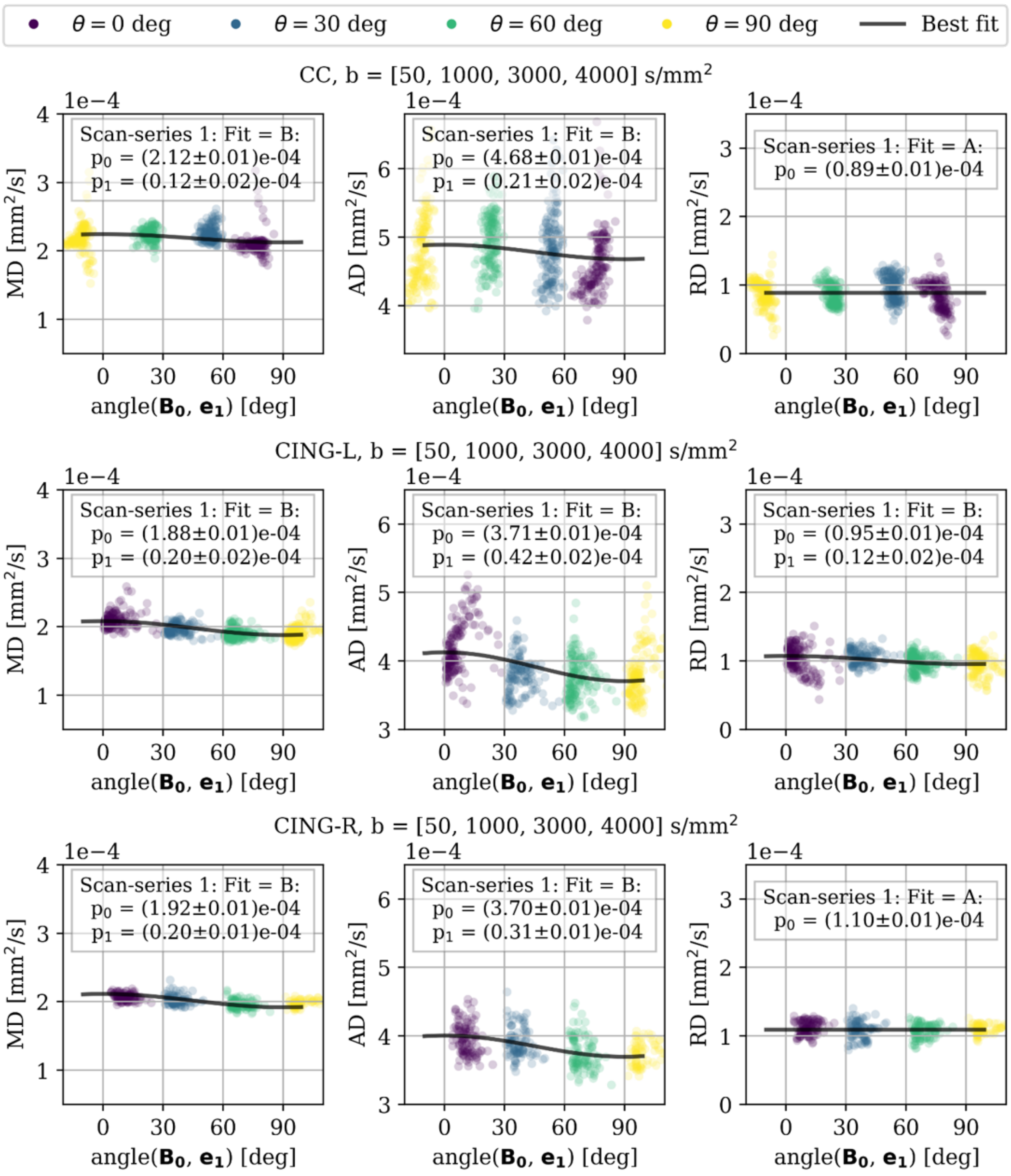

Figure S2: Scan-series 1. DT metrics fitted at low b-values ([50, 1000, 3000, 4000] s/mm^2) for CC (upper row), CING-L (middle row), and CING-R (lower row) of an ex vivo monkey brain at 7 T. Markers are coloured according to which orientation the scan was acquired at, and plotted along with the best fit based on lowest AIC-value. Orientation-dependence is observed for AD of all ROIs, and for MD of CC. The degree of orientation-dependence is stronger for CC than for CING-L and CING-R. The difference in effect on AD vs. RD shows that the PGSE signal is affected anisotropically. ΔAIC and ΔRMSE for all DT metrics and all fitted models are listed in Tab. S1

ΔAIC for CC			
Model\Metric	MD	AD	RD
A	11	12	**0**
B	**0**	**0**	12
C	34	16	16
D	33	15	15

ΔAIC for CING-L			
Model\Metric	MD	AD	RD
A	284	111	27
B	**0**	**0**	**0**
C	286	87	55
D	320	114	59

ΔAIC for CING-R			
Model\Metric	MD	AD	RD
A	64	75	**0**
B	**0**	**0**	60
C	108	84	13
D	133	102	35

Table S1: Fitting performance according to ΔAIC for orientation-dependence of DT metrics of scan-series 1 at low b-values ([50, 1000, 3000, 4000] s/mm^2) for CC (upper row), CING-L (middle row), and CING-R (lower row).

ΔAIC for CC			
Model\Metric	MD	AD	RD
A	143	116	**0**
B	**0**	**0**	8
C	112	112	12
D	151	119	22

ΔAIC for CING-L			
Model\Metric	MD	AD	RD
A	17	32	**0**
B	**0**	**0**	28
C	89	39	44
D	80	39	26

ΔAIC for CING-R			
Model\Metric	MD	AD	RD
A	21	50	**0**
B	**0**	**0**	36
C	98	57	46
D	93	57	27

Table S2: Fitting performance according to ΔAIC for orientation-dependence of DT metrics of scan-series 2 at low b-values ([50, 1000, 3000, 4000] s/mm^2) for CC (upper row), CING-L (middle row), and CING-R (lower row).

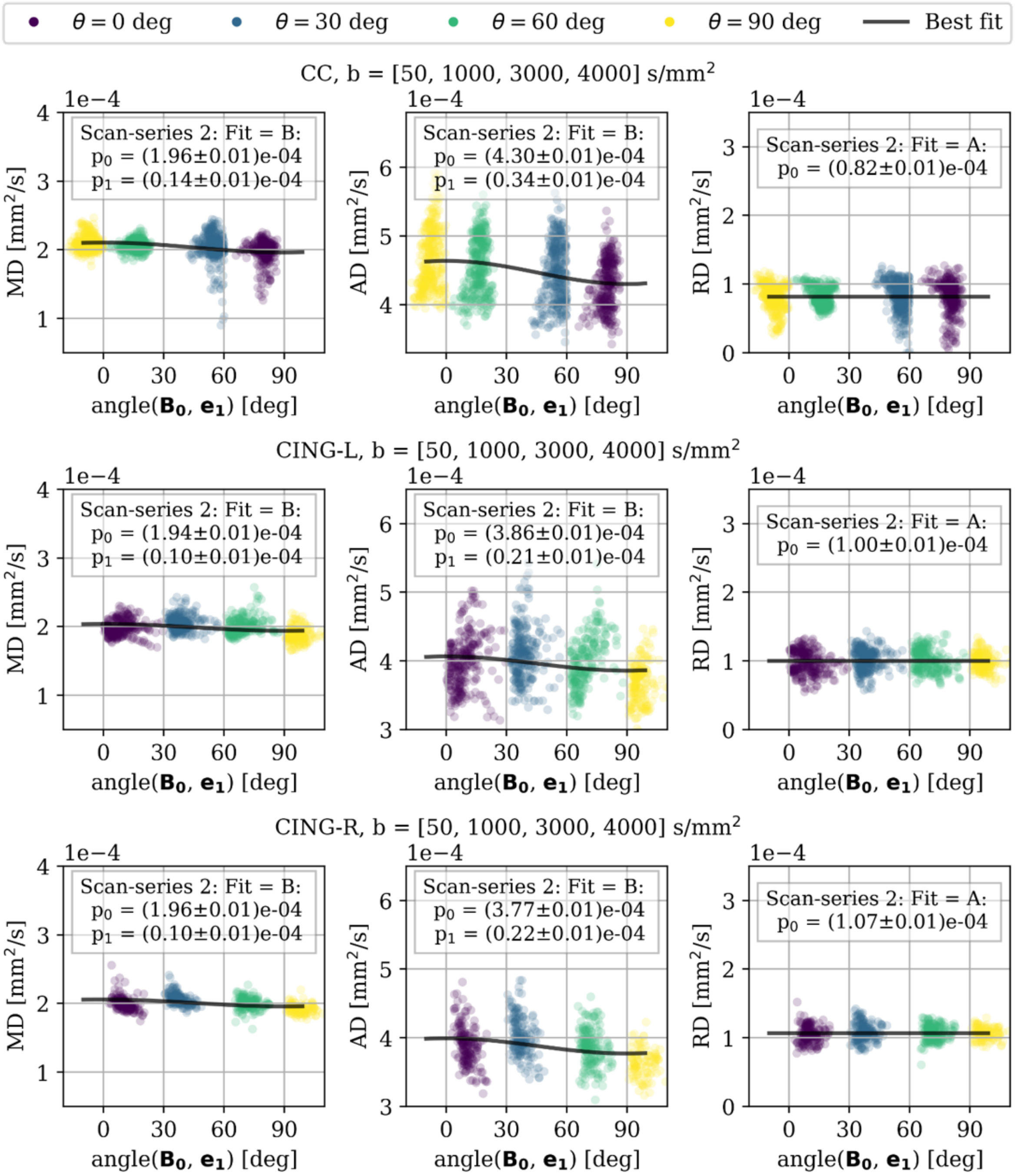

Figure S3: Scan-series 2. DT metrics fitted at low b-values ([50, 1000, 3000, 4000] s/mm^2) for CC (upper row), CING-L (middle row), and CING-R (lower row) of an ex vivo monkey brain at 7 T. Markers are coloured according to which orientation the scan was acquired at, and plotted along with the best fit based on lowest AIC-value. Orientation-dependence is observed for AD of all ROIs, and for MD of CC and CING-L. The degree of orientation-dependence is stronger for CC than for CING-L and CING-R. The difference in effect on AD vs. RD shows that the PGSE signal is affected anisotropically. ΔAIC and ΔRMSE for all DT metrics and all fitted models are listed in Tab. S2

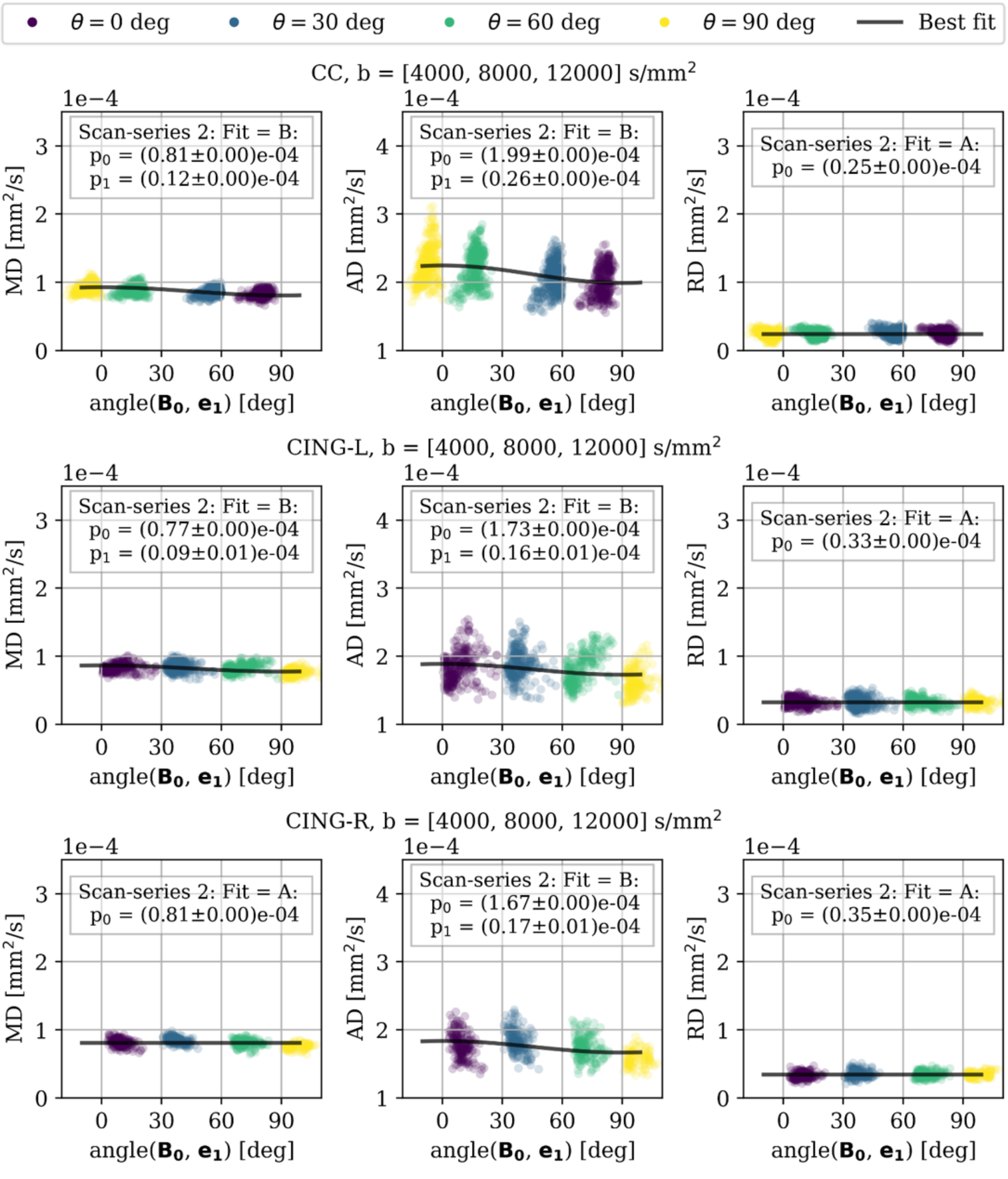

Figure S4: Scan-series 2. DT metrics fitted at intermediate b-values ([4000, 8000, 12000] s/mm^2) for CC (upper row), CING-L (middle row), and CING-R (lower row) of an ex vivo monkey brain at 7 T. Markers are coloured according to which orientation the scan was acquired at, and plotted along with the best fit based on lowest AIC-value. Orientation-dependence is observed for AD of all ROIs, and for MD of CC. The degree of orientation-dependence is stronger for CC than for CING-L and CING-R. The difference in effect on AD vs. RD shows that the PGSE signal is affected anisotropically. ΔAIC and ΔRMSE for all DT metrics and all fitted models are listed in Tab. S3

ΔAIC for CC			
Model\Metric	MD	AD	RD
A	204	181	**0**
B	**0**	**0**	95
C	197	149	106
D	437	190	210

ΔAIC for CING-L			
Model\Metric	MD	AD	RD
A	7	49	**0**
B	**0**	**0**	124
C	200	71	163
D	183	67	145

ΔAIC for CING-R			
Model\Metric	MD	AD	RD
A	**0**	67	**0**
B	1	**0**	162
C	189	89	212
D	170	86	141

Table S3: Fitting performance according to ΔAIC for orientation-dependence of DT metrics of scan-series 2 at intermediate b-values ([4000, 8000, 12000] s/mm^2) for CC (upper row), CING-L (middle row), and CING-R (lower row).

ΔAIC for CC			
Model\Metric	MD	AD	RD
A	280	212	**0**
B	**0**	**0**	327
C	252	151	403
D	622	237	895

ΔAIC for CING-L			
Model\Metric	MD	AD	RD
A	**0**	33	**0**
B	126	**0**	594
C	363	79	734
D	323	74	601

ΔAIC for CING-R			
Model\Metric	MD	AD	RD
A	**0**	71	**0**
B	149	**0**	436
C	386	122	502
D	341	116	322

Table S4: Fitting performance according to ΔAIC for orientation-dependence of DT metrics of scan-series 2 at high b-values ([8000, 12000, 20000] s/mm^2) for CC (upper row), CING-L (middle row), and CING-R (lower row).

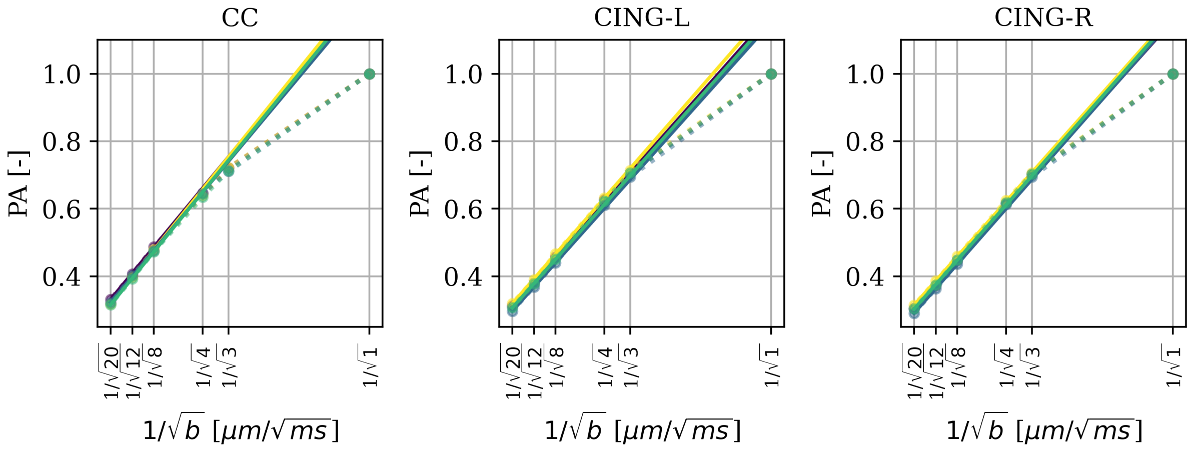

Figure S5: Validation of the signal in our high b-value regime (b=[8, 12, 20] ms/μm^2) expressing an isolated intra-axonal compartment by the powder average (PA) following a $1/\sqrt{b}$ law [1][2]. Solid lines are linear fits over b=[8, 12, 20] ms/μm^2. With R2 $\geq$ 0.999 for all fits.

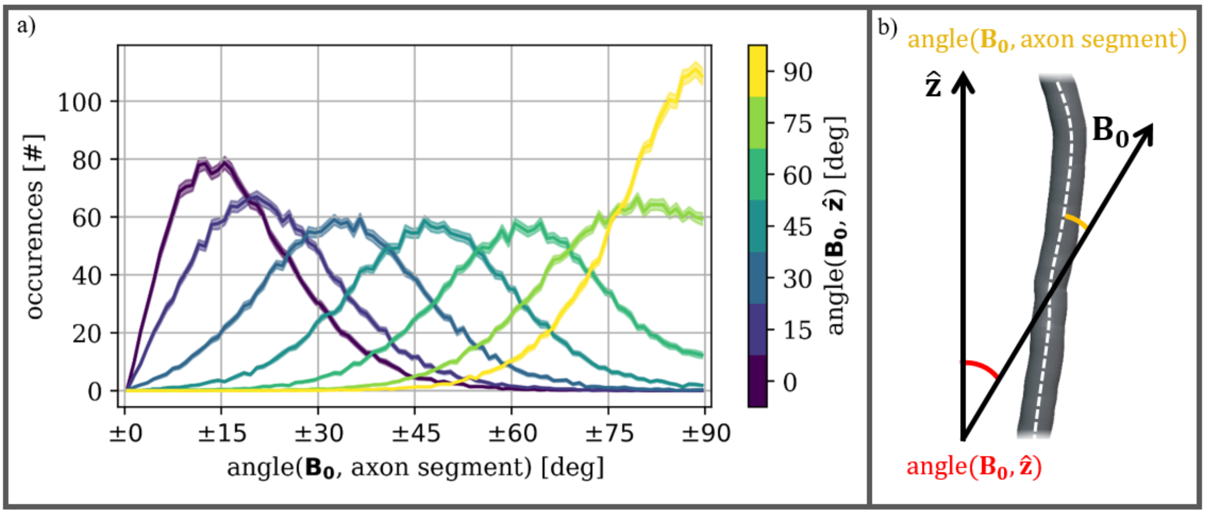

Figure S6: **a)** Distributions of angles between $\mathbf{B_0}$ and longitudinal axon segments of C3, as they appear at different orientations between $\mathbf{B_0}$ and the overall orientation of the axons $\hat{\mathbf{z}}$. Lines are the mean distribution over all 29 C3 axons. Margins are means $\pm$ std/$\sqrt{N=29}$. **b)** Illustration of how the angles in a) are measured for the C3 axons.

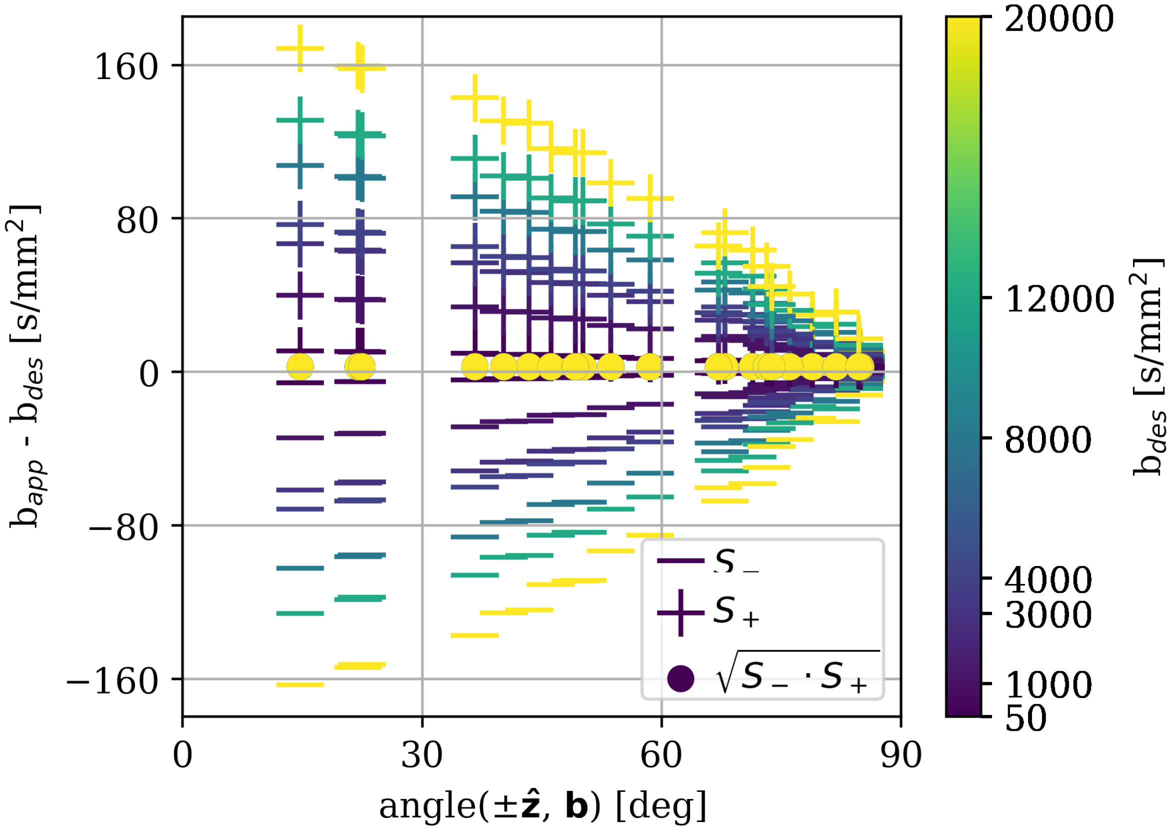

Figure S7: Cross-term correction of the applied b-values. For 2D imaging the applied b-values become skewed along the image-slice direction due to the super-position of slice gradients and diffusion gradients. +'s indicate the deviation between the applied b-value and the desired b-value for b-vectors with a positive component along $\hat{\mathbf{z}}$. Vice versa for -'s. ·'s indicate the cross-term corrected b-value. For increasing b-values we see increasing anisotropy for the uncorrected values (i.e. the deviation of the magnitude of b-vectors at different orientations increases).

References

1. Jensen, J. H., Russell Glenn, G & Helpern, J. A. Fiber ball imaging. en. *Neuroimage* **124,** 824–833 (Jan. 2016).
2. Veraart, J., Fieremans, E. & Novikov, D. S. On the scaling behavior of water diffusion in human brain white matter. en. *Neuroimage* **185,** 379–387 (Jan. 2019).
